# Suppression of SARS-CoV-2 nucleocapsid protein dimerization by ISGylation and its counteraction by viral PLpro

**DOI:** 10.3389/fmicb.2024.1490944

**Published:** 2024-10-24

**Authors:** Wonjin Bang, Jaehyun Kim, Kanghun Seo, Jihyun Lee, Ji Ho Han, Daegyu Park, Jae Hwan Cho, Donghyuk Shin, Kyun-Hwan Kim, Moon Jung Song, Jin-Hyun Ahn

**Affiliations:** ^1^Department of Microbiology, Sungkyunkwan University School of Medicine, Suwon, Republic of Korea; ^2^Department of Biotechnology, College of Life Sciences and Biotechnology, Korea University, Seoul, Republic of Korea; ^3^Department of Systems Biology, College of Life Science and Biotechnology, Yonsei University, Seoul, Republic of Korea; ^4^Department of Precision Medicine, Sungkyunkwan University School of Medicine, Suwon, Republic of Korea

**Keywords:** SARS-CoV-2, ISG15, nucleocapsid, interferon, PLpro

## Abstract

Protein modification by the ubiquitin-like protein ISG15 (ISGylation) plays a crucial role in the immunological defense against viral infection. During severe acute respiratory syndrome coronavirus 2 (SARS-CoV-2) infection, innate immune signaling proteins are ISGylated, facilitating innate immunity. However, whether SARS-CoV-2 proteins are direct substrates for ISGylation remains unclear. In this study, we investigated whether SARS-CoV-2 proteins undergo ISGylation and whether ISGylation affects viral protein function. Co-transfection ISGylation analysis of SARS-CoV-2 proteins showed that the nucleocapsid (N) protein is ISGylated at several sites. Herc5 promoted N ISGylation and interacted with N, indicating that Herc5 acts as an E3 ligase for N ISGylation. Lys-261 (K261) within the oligomerization domain of N was identified as a potential ISGylation site that is necessary for efficient ISGylation of N. K261 is positioned at the center of the dimer interface in the crystal structure of the C-terminal domain dimer and the ISGylated form of N showed reduced protein dimerization in pull-down analysis. Importantly, a recombinant virus expressing K261R mutant N showed enhanced resistance to interferon-β treatment compared to its parental virus. We also found that viral PLpro removes conjugated ISG15 from N. Our findings demonstrate that ISGylation of SARS-CoV-2 N inhibits protein dimerization, resulting in viral growth more susceptible to type I interferon responses, and that viral PLpro counteracts this ISG15-mediated antiviral activity by removing conjugated ISG15 from N.

## Background

ISG15 is a ubiquitin-like protein encoded by interferon (IFN)-stimulated gene 15. Like ubiquitin conjugation, ISG15 can be conjugated to proteins via an E1, E2, and E3 enzymatic cascade. For ISG15 conjugation (termed ISGylation) in humans, UBE1L acts as the E1-activating enzyme, and UbcH8 is the E2-conjugating enzyme. Herc5, EFP/TRIM25, and HHARI are E3 ligases. ISGylation is a reversible process. The ubiquitin-specific protease USP18/UBP43 can act as a deISGylase, removing conjugated ISG15 from substrates. All ISGylation and deISGylation enzymes are IFN-inducible (Kang et al., 2022). Independent of deISGylating activity, USP18 can negatively regulate innate immune responses by binding IFNAR2, a type I IFN receptor subunit (Malakhova et al., 2006; Arimoto et al., 2017). ISG15 can also be secreted from the cell, where it has several immunomodulatory and cytokine-like functions (Perng and Lenschow, 2018; Swaim et al., 2020).

As Herc5 is associated with polyribosomes, ISGylation broadly affects protein functions, and newly synthesized viral proteins may be the main target of ISG15 in virus-infected cells (Durfee et al., 2010). ISG15 and ISGylation repress the growth of diverse RNA and DNA viruses. ISGylation inhibits various steps of the virus life cycle, including viral entry, gene expression, genome replication, and virion maturation and release, by directly modulating the function of viral proteins or regulating cellular proteins (Morales and Lenschow, 2013; Sarkar et al., 2023). In contrast, the proviral activity of ISG15 has also been demonstrated. In humans, free ISG15 promotes sustained expression of USP18, which negatively regulates IFN signaling (Bogunovic et al., 2012; Zhang et al., 2015; Meuwissen et al., 2016; Speer et al., 2016). Not surprisingly, many viruses have evolved ways to regulate ISG15 pathways (Perng and Lenschow, 2018; Sarkar et al., 2023).

Severe acute respiratory syndrome coronavirus 2 (SARS-CoV-2) is the causative agent of the coronavirus disease 2019 (COVID-19) pandemic (Zhou P. et al., 2020; Zhu et al., 2020). Like other coronaviruses, SARS-CoV-2 is an enveloped virus containing a positive-sense single-strand RNA genome of ~30 kb (Lu et al., 2020; Wu et al., 2020). SARS-CoV-2 encodes 16 non-structural proteins (NSPs), four structural proteins, and several accessory proteins. The NSPs are produced from genomic RNA and play an essential role in viral transcription and replication. The structural and accessory proteins are produced from subgenomic RNAs. The structural proteins include spike (S), envelope (E), membrane (M), and nucleocapsid (N) proteins, which surround and protect the RNA genome and play an essential role in replication and virion assembly. Accessory proteins, of which there are at least six, are important virulence factors in SARS-CoV-2 infection (Kim et al., 2020; V'kovski et al., 2021).

Studies on the regulation of ISG15 pathways during SARS-CoV-2 infection have focused on the deISGylase activity of the viral papain-like protease (PLpro), an essential enzyme required for processing viral polyproteins. Retinoic-acid inducible gene I (RIG-I)-like receptors, such as RIG-I and melanoma differentiation-associated protein 5 (MDA5), are cytoplasmic RNA sensors that recognize viral and host-immunostimulatory RNAs to induce innate immunity (Schlee, 2013). SARS-CoV-2 PLpro preferentially cleaves ISG15, whereas SARS-CoV PLpro (sharing 83% sequence identity) predominantly removes ubiquitin. Indeed, ISG15 cleavage from IFN regulatory factor 3 (IRF3) by PLpro attenuates type I INF responses (Shin et al., 2020). ISGylation of MDA5 promotes its oligomerization and triggers the activation of innate immunity, and ISG15-dependent activation of MDA5 is also antagonized by PLpro, which cleaves ISG15 from MDA5 (Liu et al., 2021). Compared to Zika and influenza viruses, SARS-CoV-2 infection in human macrophages results in very low levels of ISGylated proteins with free ISG15 accumulation due to the PLpro activity, which also correlates with macrophage polarization toward a pro-inflammatory phenotype, and promotes ISG15 secretion via unconventional secretory pathways (Munnur et al., 2021). Therefore, the deISGylating activity of PLpro plays an important role in SARS-CoV-2 pathogenesis.

Although the regulatory role of PLpro in ISGylation of cellular proteins in SARS-CoV-2 infection has been demonstrated, whether viral proteins are ISGylated and whether this affects protein function remain unclear. In this study, we show that the N protein of SARS-CoV-2 is ISGylated, inhibiting protein dimerization. The effect of N ISGylation on viral growth over host IFN responses is analyzed using a recombinant virus that mutates a residue necessary for efficient ISGylation. We also show that PLpro removes conjugated ISG15 from N, indicating that SARS-CoV-2 PLpro prevents ISG15-mediated antiviral responses acting on essential viral proteins.

## Methods

### Cells and viruses

Human embryonic kidney 293 T, immortalized human epithelial HeLa, African green monkey kidney epithelial Vero, and human lung adenocarcinoma Calu-3 and A549 cell lines were grown in Dulbecco’s modified eagle medium (DMEM) (Sigma-Aldrich), supplemented with 10% fetal bovine serum and 100 U of penicillin–streptomycin (Gibco) in a 5% CO_2_ humidified incubator at 37°C. DNA transfection of cells was performed using the polyethyleneimine (PEI) version of the cationic polymer procedure. The recombinant viruses expressing either wild-type N or mutant N(K261R) were produced using the seven plasmid-based reverse genetic system of SARS-CoV-2 (Xie et al., 2020; Xie et al., 2021), following the site-directed mutagenesis on the N gene of F7 plasmid. All the experiments using recombinant SARS-CoV-2 viruses were conducted in a Bio Safety Level 3 facility.

### Plaque assays

SARS-CoV-2 recombinant viruses were reconstituted and propagated in Vero cells, and the virus titer was determined by plaque assays. Briefly, Vero cells were inoculated with a 10-fold serially diluted stock of SARS-CoV-2. After 1 h, the inoculum was discarded, and the cells were overlaid with DMEM-F12 (Sigma-Aldrich) containing 2% agarose and 1% penicillin–streptomycin. After 3–4 days, the cells were stained with 0.2% crystal violet solution (20% ethanol) overnight. Plaques were counted in duplicates of each sample, and the virus titer was calculated.

### Expression plasmids

Twenty-six SARS-CoV-2 open-reading frames (ORFs) in pENTR clones were purchased from Addgene. SARS-CoV-2 ORFs were cloned into the pCS3-MT (with a 6×Myc tag)-based destination vector (Turner and Weintraub, 1994) using LR clonase (Invitrogen). Plasmids expressing ISG15_GG_, an active form of ISG15 with a termination codon added immediately after the double glycine residues, or ISG15_AA_, a conjugation-defective mutant in which the double glycine residues are replaced with alanine residues, and the pSG5-driven plasmids expressing Flag-UbcH8 and HA-Herc5 were as described previously (Kim et al., 2016). Plasmids for HA-UBE1L (pCAGGS-HA-hUBE1L) were provided by Dong-Er Zhang (University of California San Diego). The pCMV6 vectors expressing SRT-ISG15_GG_ and SRT-N were produced using Gateway technology. Flag-EFP (Plasmid #12449) and Flag-HHARI (Plasmid #17450) plasmids were purchased from Addgene. The pEXP-CS3-MT-N plasmids expressing truncated mutants of N (80–419, 200–419, 270–419, 1–365, and 1–200) were made using PCR. The lysine-to-arginine mutant N constructs were produced by site-direct mutagenesis (Stratagene QuikChange). Plasmids expressing Flag-PLpro and Flag-PLpro (C111A) were as described previously (Shin et al., 2020). Plasmids containing Myc-UBP43 were created by transferring UBP43 cDNA into a pCS3-MT-based destination vector using LR clonase (Invitrogen).

### Antibodies

Anti-HA rat monoclonal antibody (MAb, 3F10) and anti-Myc mouse MAb (9E10) conjugated with peroxidase were purchased from Roche. Mouse MAb against ISG15 (F-9) was obtained from Santa Cruz. Mouse MAb against the SRT epitope was as described previously (Lee et al., 2003). Mouse MAb against β-actin was purchased from Sigma. Anti-S (ab272504) rabbit polyclonal Ab (PAb) and anti-N (40143-R019) rabbit MAb were purchased from Abcam and Sino Biological, respectively.

### Immunoblot assay

Cells were washed with cold phosphate-buffered saline (PBS), and total cell lysates were prepared by boiling in sodium dodecyl sulfated (SDS) loading buffer. Equal amounts of the cell lysates were separated by SDS-polyacrylamide gel electrophoresis (PAGE), and then the protein was transferred onto a nitrocellulose membrane (GE Healthcare). The membrane was blocked for 1 h or more in PBS plus 0.1% Tween 20 (PBST, Sigma) containing 5% skim milk and washed with PBST. After incubation with the appropriate antibody, the protein was visualized using an enhanced chemiluminescence system (Roche) and X-ray film. ImageJ (NIH) was used to quantify the relative protein levels in immunoblots.

### Co-immunoprecipitation (co-IP) assays

Two days after transfection, 293 T cells (8 × 10^5^) were harvested and sonicated in 0.7 mL co-IP buffer (50 mM Tris-Cl, pH 7.4, 50 mM NaF, 5 mM sodium phosphate, 0.1% Triton X100, containing protease inhibitors, Sigma) by a microtip probe (Vibra-Cell; Sonics and Materials, Inc.) for 10 s (pulse on l s, pulse off 3 s). Cell lysates were incubated with appropriate antibodies. After incubation for 16 h at 4°C, 30 μL of a 50% slurry of protein A and G-Sepharose (Amersham) were added, and then the mixture was incubated for 2 h at 4°C to allow adsorption. The mixture was then pelleted and washed seven times with co-IP buffer. The beads were resuspended and boiled for 5 min in a loading buffer. Each sample was analyzed by SDS-PAGE and immunoblotted with appropriate antibodies.

### Purification of 6×His-N proteins in bacterial cells

*Escherichia coli* BL21(DE3) cells harboring the plasmids expressing N proteins were cultured in LB broth at 37°C until the optical density at 600 nm (OD600) reached 0.4–0.6. The expression of N protein was induced by the addition of 0.5 mM IPTG for 4 h. Cells were harvested by centrifugation at 6,000 × *g* for 7 min at 4°C, resuspended in buffer A (50 mM Tris–HCl pH 8.0, 0.5 M NaCl, and 40 mM imidazole), and disrupted by sonication on ice. After centrifugation at 48,000 × *g* for 1 h at 4°C, the cell-free extracts were loaded onto a 5-mL Ni^2+^–NTA column (GE Healthcare), and bound proteins were eluted by a gradient of 0.04–1.0 M imidazole in buffer A. The elution fractions containing N protein were subjected to buffer exchange in PBS by dialysis. Proteins were then concentrated and stored at −80°C until use.

### Pull-down assays with 3′ biotin-tagged nucleic acids

Nucleic acids were immobilized with 30 μg of streptavidin agarose beads (Sigma, S1638) in 300 μL of buffer (10 mM Tris, pH 7.5, RT, 1 mM EDTA, 1 M NaCl, 0.003% NP40) for 30 min at room temperature with constant rotation. The immobilized nucleic acids were collected by centrifugation and incubated in 300 μL of blocking buffer [2.5 mg/mL of BSA in 10 mM HEPES, pH 7.6, RT, 100 mM potassium glutamate, 2.5 mM dithiothreitol (DTT), 10 mM magnesium acetate, 5 mM EGTA, 3.5% glycerol with 0.003% NP40, and 5 mg/mL of polyvinylpyrrolidone] for 30 min at room temperature to minimize non-specific interactions. The immobilized nucleic acids were collected using centrifugation and incubated with 1 μg of purified 6×His-N in 400 μL of binding buffer (10 mM HEPES, pH 7.6, RT, 100 mM potassium glutamate, 80 mM KCl, 2.5 mM DTT, 10 mM magnesium acetate, 5 mM EGTA, 3.5% glycerol with 0.002% NP40, and 1 μg of non-specific carrier DNA) for 1 h at 4°C with constant rotation. After 1 h incubation, the immobilized nucleic acid/protein complexes were washed three times with 500 μL of washing buffer (10 mM HEPES, pH 7.6, RT, 100 mM potassium glutamate, 2.5 mM DTT, 10 mM magnesium acetate, 5 mM EGTA, 3.5% glycerol, 0.5 mg/mL BSA, and 0.2% NP40). Twenty μL of SDS–polyacrylamide gel electrophoresis (PAGE) sample buffer was added to the immobilized nucleic acid/protein complexes and incubated at 37°C for 15 min. After centrifugation, the supernatants (eluents) were boiled at 97°C for 7 min. Nucleic acid bound 6×His-N were detected by immunoblot assay using anti-N antibody (33,717, CST).

### N protein pull-down assays

The 293T cells transfected with 6×Myc-N(200–419) plasmid were treated with 5 mM N-ethylmaleimide (NEM) for 30 min before they were harvested. Cell pellets were resuspended with 2% SDS lysis buffer containing protease inhibitors (Sigma) and boiled for 10 min. Cell lysates were diluted 10-fold with co-IP buffer and sonicated using a Vibra-Cell microtip probe (Sonics and Materials) for 10 s (pulse on 1 s, pulse off 3 s). The clarified cell lysates were pre-cleared for 2 h with 30 μL of a 50% slurry of Ni^2+^–NTA His∙Bind Resin (Novagen). After pre-clearing, the clarified cell lysates were incubated for 16 h with the bacterial purified 6×His-N protein at 4°C overnight. Then, 30 μL of 50% Ni^2+^–NTA His∙Bind Resin (Novagen) slurry was added. After incubation for 2 h at 4°C, the mixture was pelleted and washed several times with co-IP buffer. Each sample was analyzed by SDS-PAGE and immunoblotting with the appropriate antibody.

## Results

### SARS-CoV-2 N is modified by ISG15

**Figure 1:**
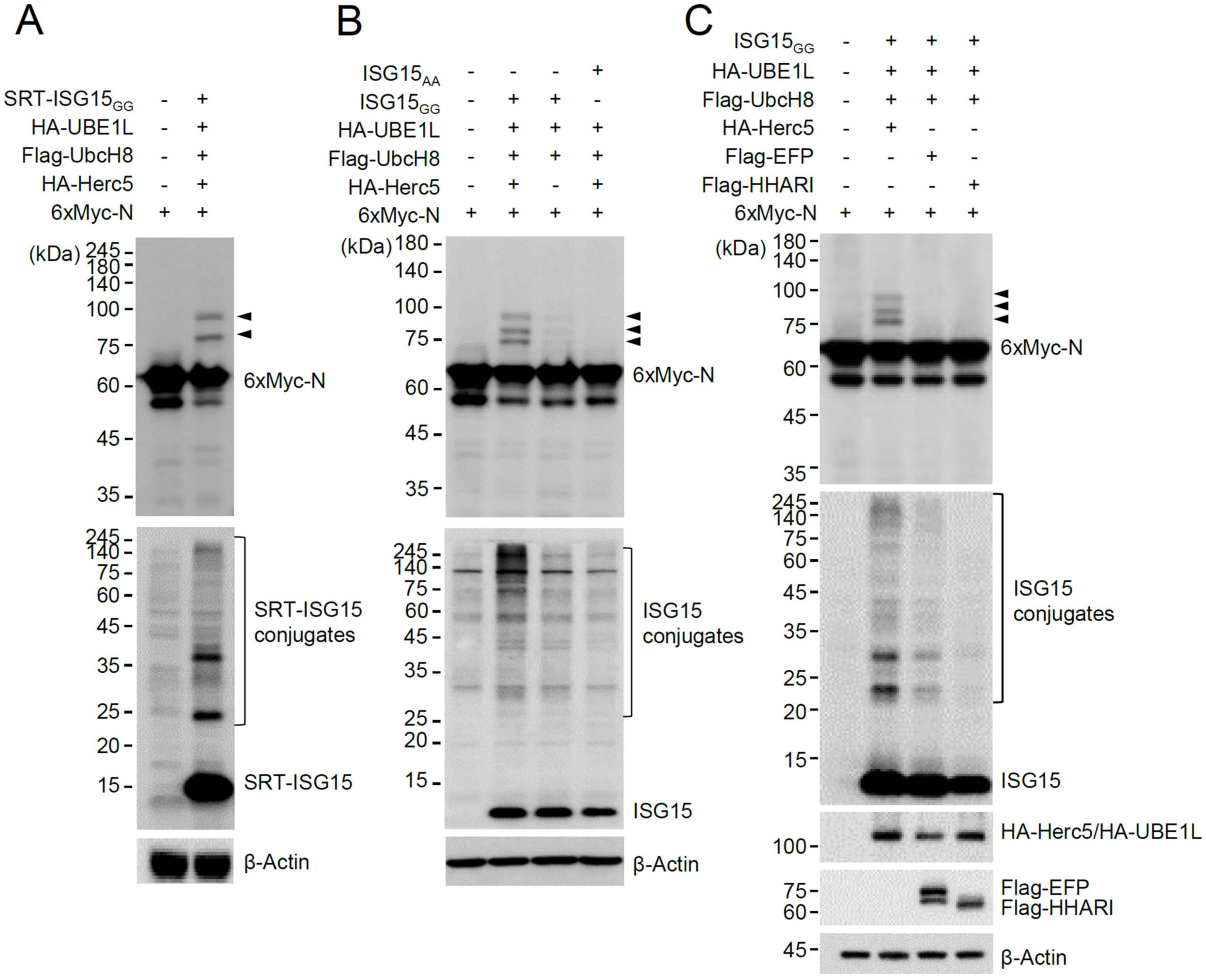
Herc5-mediated ISGylation of N. **(A,B)** Plasmids expressing 6×Myc-N (0.25 μg), HA-UBE1L (0.2 μg), Flag-UbcH8 (0.2 μg), HA-Herc5 (0.4 μg), and SRT-ISG15_GG_ (0.2 μg) **(A)** or ISG15_GG_ or ISG15_AA_ (0.2 μg) **(B)** were co-transfected into 293T cells in six-well plates as indicated. At 48 h after transfection, total cell lysates were prepared and subjected to SDS-PAGE, followed by immunoblot assays with anti-Myc and anti-SRT **(A)** or anti-Myc and anti-ISG15 antibodies **(B)**. β-actin was used as a loading control. The positions of N proteins with one and two SRT-ISG15 moieties **(A)** or one to three ISG15 moieties **(B)** are indicated (arrowheads). **(C)** Plasmids expressing 6×Myc-N, HA-UBE1L, Flag-UbcH8, ISG15_GG_ and one of three E3 ligases (HA-Herc5, Flag-EFP, or Flag-HHARI) (0.4 μg) were co-transfected into 293T cells in six-well plates as indicated. At 48 h after transfection, total cell lysates were prepared and subjected to SDS-PAGE, followed by immunoblot assays with anti-Myc, anti-ISG15, anti-HA, anti-Flag and anti-β-Actin antibodies. The positions for ISGylated N proteins are indicated (arrowheads).

To investigate whether SARS-CoV-2-encoded proteins are modified by ISG15, we produced mammalian expression vectors that express 6×Myc-tagged SARS-CoV-2 proteins and performed co-transfection ISGylation assays using plasmids expressing SRT-ISG15_GG_ (an active form of ISG15), HA-UBE1L (E1), Flag-UbcH8 (E2), and HA-Herc5 (E3). Among 27 viral proteins tested, the N protein was repeatedly identified as a prominent ISGylation substrate in 293T cells overexpressing ISG15 and ISGylation enzymes (Supplementary Figure S1). We observed two forms of modified N proteins with either one or two SRT-ISG15 moieties (Figure 1A, arrowheads).

ISGylation of N was also assessed using untagged ISG15. In similar co-transfection assays, using untagged ISG15_GG_ and 6×Myc-N produced three different ISG15-modified N proteins that contain one to three ISG15 moieties (Figure 1B, arrowheads). ISGylation of N was greatly diminished when Herc5 was omitted, or ISG15_AA_ (an inactive form) was used (Figure 1B), demonstrating that N is a true substrate for ISGylation. When the human E3 ligases involved in ISGylation (Herc5, EFP, and HHARI) were compared, Herc5 and EFP effectively increased ISGylation under given conditions compared to HHARI, but only Herc5 enhanced the ISGylation of N, suggesting that Herc5 acts as the primary E3 ligase for N ISGylation (Figure 1C).

### N interacts with Herc5 and K261 of N is necessary for efficient ISGylation

**Figure 2:**
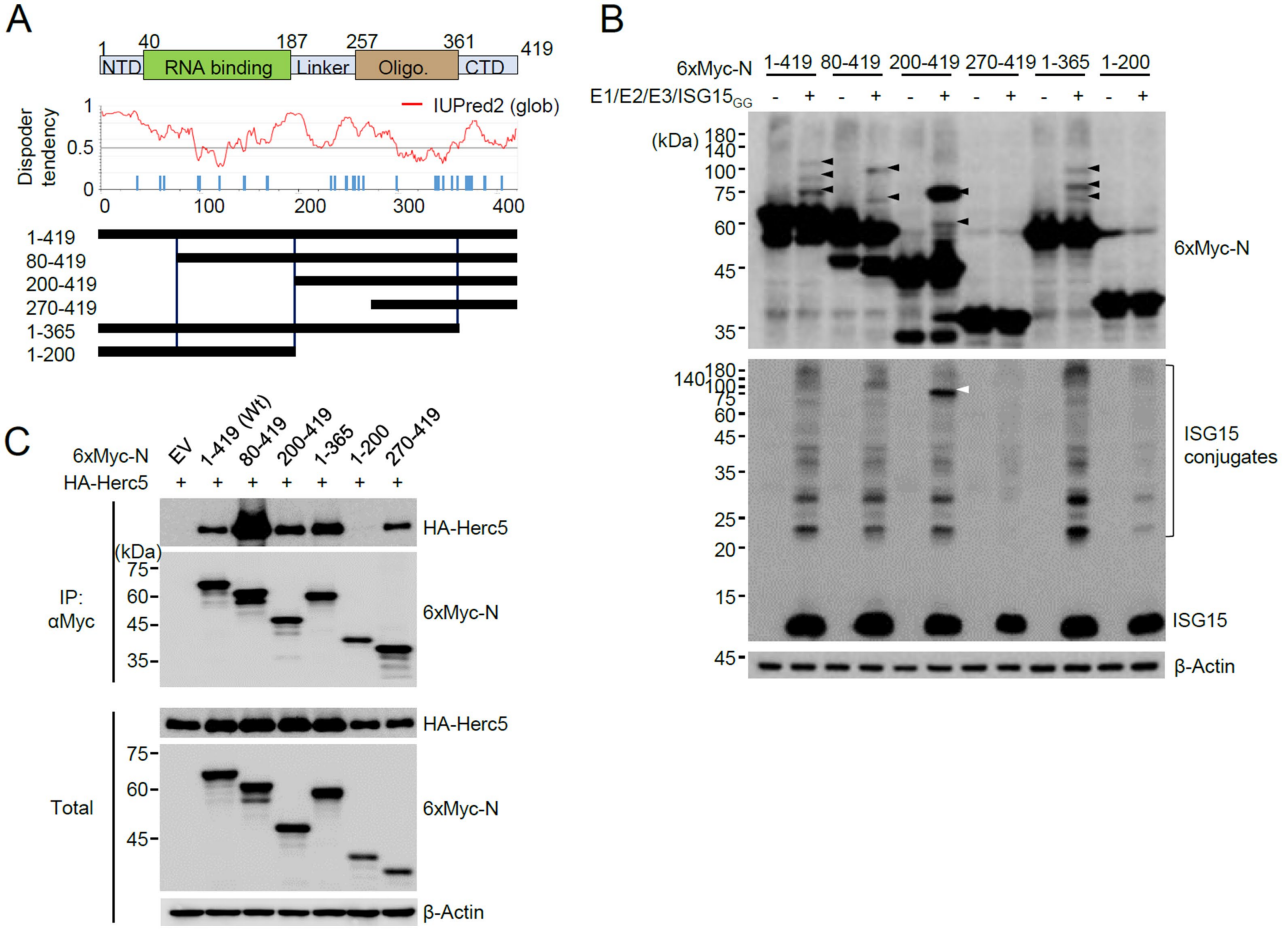
Identification of the N domains required for ISGylation. **(A)** RNA binding and oligomerization domains of N are indicated at the top. NTD, N-terminal domain; CTD, C-terminal domain. The amino acid positions are numbered. The disorder tendency of N analyzed by IUPred2 is shown in the middle. Wild-type N and truncated N constructs were based on disorder tendencies and the positions of lysine residues. **(B)** Plasmids expressing 6×Myc-N or its truncated forms (0.25 μg), HA-UBE1L (E1, 0.2 μg), Flag-UbcH8 (E2, 0.2 μg), HA-Herc5 (E3, 0.4 μg), and ISG15_GG_ (0.2 μg) were co-transfected into 293T cells in six-well plates. At 48 h after transfection, total cell lysates were prepared and subjected to SDS-PAGE, followed by immunoblot assays with anti-Myc, anti-ISG15, and anti-β-Actin antibodies. **(C)** Empty vector (EV) or plasmids expressing 6×Myc-N (wide-type or truncation mutants) (0.25 μg) and HA-Herc5 (0.75 μg) were co-transfected into 293T cells in six-well plates. At 48 h after transfection, cell lysates were prepared and immunoprecipitated with an anti-Myc antibody. The immunoprecipitated samples were analyzed by immunoblotting with anti-Myc and anti-HA antibodies. The expression levels of 6×Myc-N and HA-Herc5 proteins were determined by immunoblotting.

We next investigated the N domains in which ISGylation occurs. N is a 419 amino acids protein and contains 32 lysine residues. After considering the number of lysine residues and the preservation of the putative structured regions, we produced several constructs of the N protein with truncated amino (N) or carboxyl (C)-terminals and used them in co-transfection ISGylation assays (Figure 2A). Of the N-terminal truncated constructs, the 80–419 and 200–419 constructs were still ISGylated, but the 270–419 construct was not. Notably, the deletion of 199 amino acids (in 200–419) in the N-terminal resulted in a substantial accumulation of a modified form with two ISG15 moieties. Strong ISGylation in the 200–419 construct was also evident in western blotting with an anti-ISG15 antibody (Figure 2B, white arrowhead). Of the C-terminal truncation constructs, the 1–365 construct was ISGylated, similar to the wild-type, but 1–200 was not (Figure 2B). When the SRT-tagged N proteins were used for co-transfection assays, a single ISG15-modified form of N was detected and the 270–419 and 1–200 constructs were not modified by ISG15, which is consistent with the results with 6×Myc-N (Supplementary Figure S2).

The lack of ISGylation in the 270–419 and 1–200 regions may be due to the absence of E3 binding. Therefore, we tested the interaction of wild-type and mutant N proteins with Herc5. When 293T cells were co-transfected with intact or truncated 6×Myc-N proteins and HA-Herc5 and co-IP assays were performed, the wild-type and all mutant N proteins, except 1–200, bound to Hecr5 (Figure 2C). Therefore, we could not evaluate whether the lysine residues present in the 199 amino acids of the N-terminal region act as ISG15 acceptor sites. However, this result indicated that ISG15 does not effectively modify the lysine residues of the 270–419 fragment and the Myc tag, although this fragment interacted with Herc5. The wild-type and mutant N proteins analyzed for ISGylation and Herc5 binding are indicated in Figure 2A. The 200–419 fragment is ISGylated effectively, whereas the 270–419 fragment is not. Therefore, the eight lysine residues within the 200–270 appear to be candidates for ISGylation sites.

**Figure 3:**
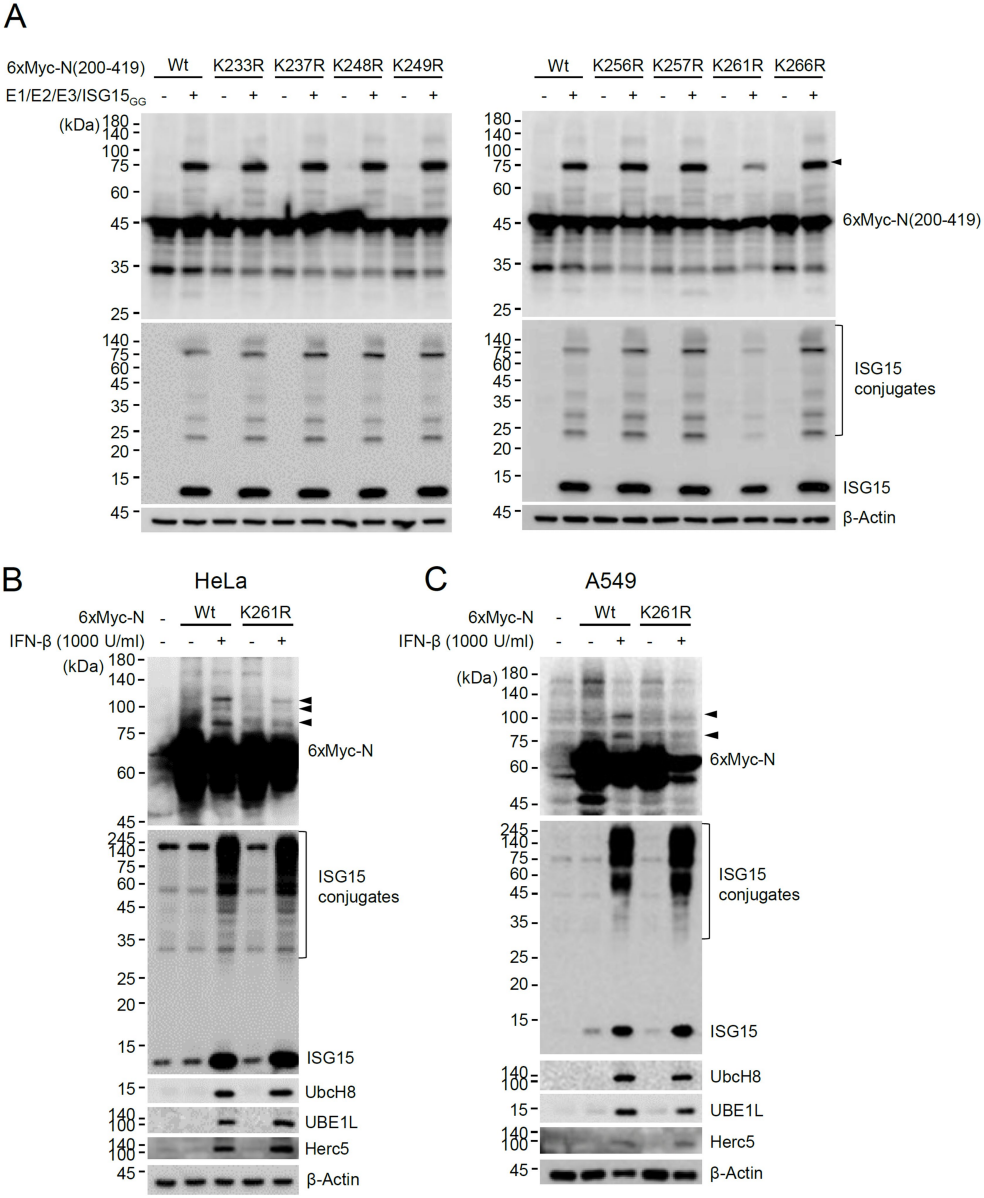
Identification of K261 as a major ISGylation site. **(A)** Plasmids expressing wild-type or K-to-R mutant 6×Myc-N(200–419) (0.25 μg), HA-UBE1L (0.2 μg), Flag-UbcH8 (0.2 μg), HA-Herc5 (0.4 μg) and ISG15_GG_ (0.2 μg) were transfected into 293T cells in six-well plates as indicated. At 48 h after transfection, total cell lysates were prepared and subjected to SDS-PAGE, and immunoblot assays were performed with anti-Myc and anti-ISG15 antibodies. The levels of β-actin are shown as a loading control. The positions for ISGylated N(200–419) protein with two ISG15 moieties are indicated (arrowhead). **(B,C)** HeLa **(B)** and A549 **(C)** cells were untreated or pretreated with IFN-β (1,000 U/mL) for 24 h and then transfected with a plasmid expressing 6×Myc-tagged N (wild-type or K261R mutant) (0.25 μg). At 24 h after transfection, cell lysates were prepared and immunoblotted with anti-Myc, anti-ISG15, anti-UBE1L, anti-UbcH8, or anti-Herc5antibodies. The levels of β-actin are shown as a loading control. The ISGylated N protein bands are indicated with arrowheads.

To investigate whether the lysine residues between 200 and 270 of N can act as an ISG15 acceptor, the 200–419 constructs containing a Lys-to-Arg mutation at 233, 237, 248, 249, 256, 257, 261, or 266 were produced, and their ISGylation was determined. The ISGylated form of 200–419 was significantly reduced by the K261R mutation, suggesting that K261 may be one of the ISGylation sites or necessary for efficient ISGylation of N (Figure 3A). The ISGylation of N was further tested in cells treated with IFN-β. When HeLa and A549 cells were pretreated with IFN-β for 24 h and then transfected with the 6×Myc-N plasmid, ISGylated forms were detected with the wild-type N, whereas they were reduced with the K261R mutant (Figures 3B,C). This result indicated that ISGylation of N occurs in IFN-β-treated cells and K261 contributes to the efficient ISGylation of N.

### ISGylation inhibits N protein dimerization

**Figure 4:**
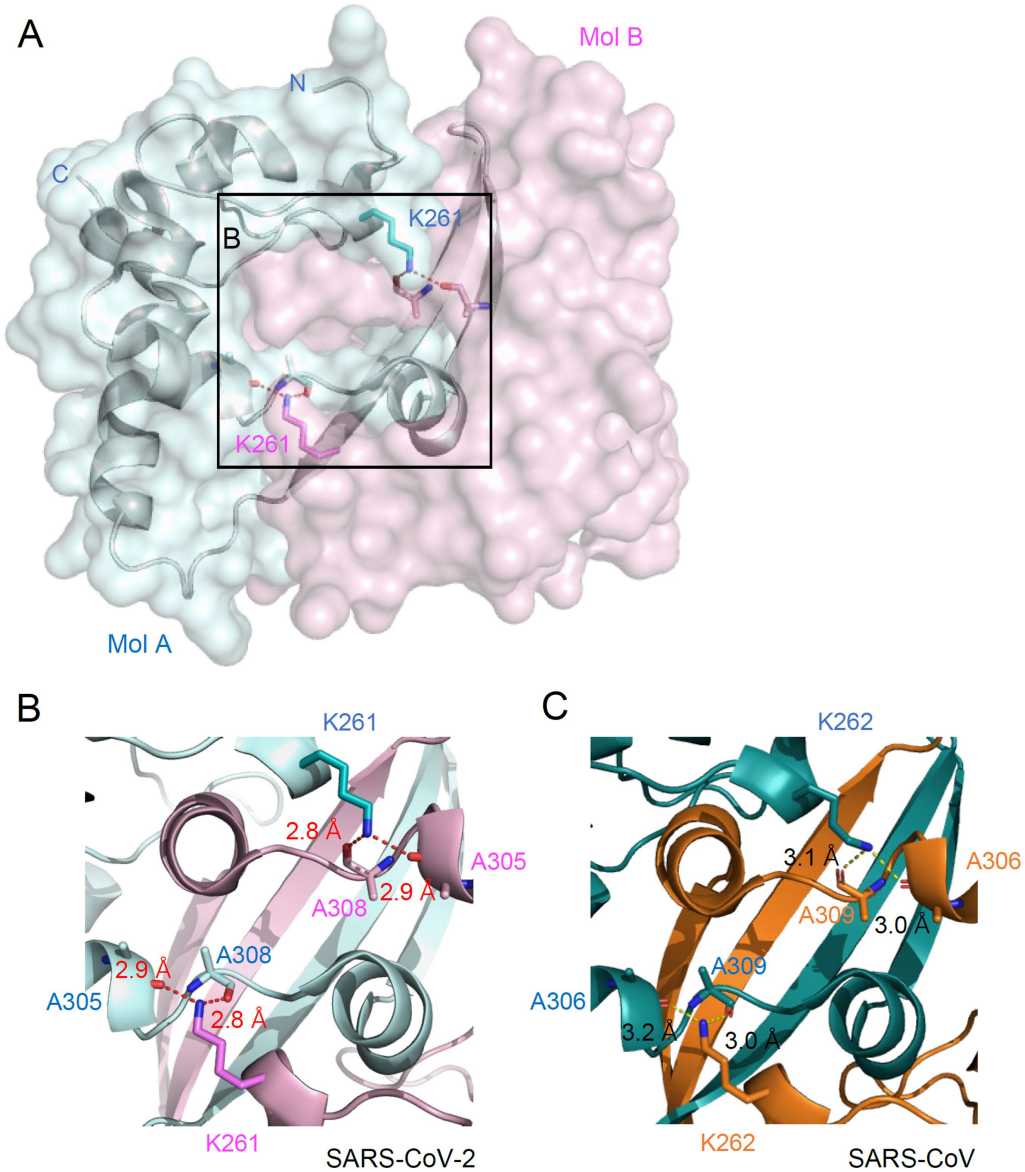
Location of K261 at the dimeric interface of N. **(A)** Overall structure of N dimer is shown in surface representation. Two N proteins are colored cyan (Mol A) and pink (Mol B). Sticks represent the K261 residue and two alanine residues. Dashed lines indicate hydrogen bonding between K261 and alanines. **(B,C)** Zoomed-in view of the dimeric interface of the N protein from SARS-CoV-2 **(B)** and SARS-CoV **(C)**. The hydrogen bonding distance is labeled.

The N proteins of SARS-CoV-2 form a dimer through a C-terminal oligomerization domain (CTD) (257–361). K261 is also located within the oligomerization domain. In the crystal structure of the N CTD dimer (PDB ID: 7VBF, 1.30 Å), K261 is located at the center of the dimer interface (Figure 4A). Specifically, K261 of one chain forms hydrogen bonds with both A305 and A308 of the other chain (Figure 4B). K261 is conserved in N proteins of all human coronaviruses. The hydrogen bonds network mediated by K261 is also found in the dimer structure of SARS-CoV (PDB ID: 2CJR, 2.50 Å), where K262 of one chain forms hydrogen bonds with both A306 and A309 of the other chain (Figure 4C). This indicates that the hydrogen bonding mediated by K261 may be involved in the oligomerization of N proteins in human coronaviruses.

**Figure 5:**
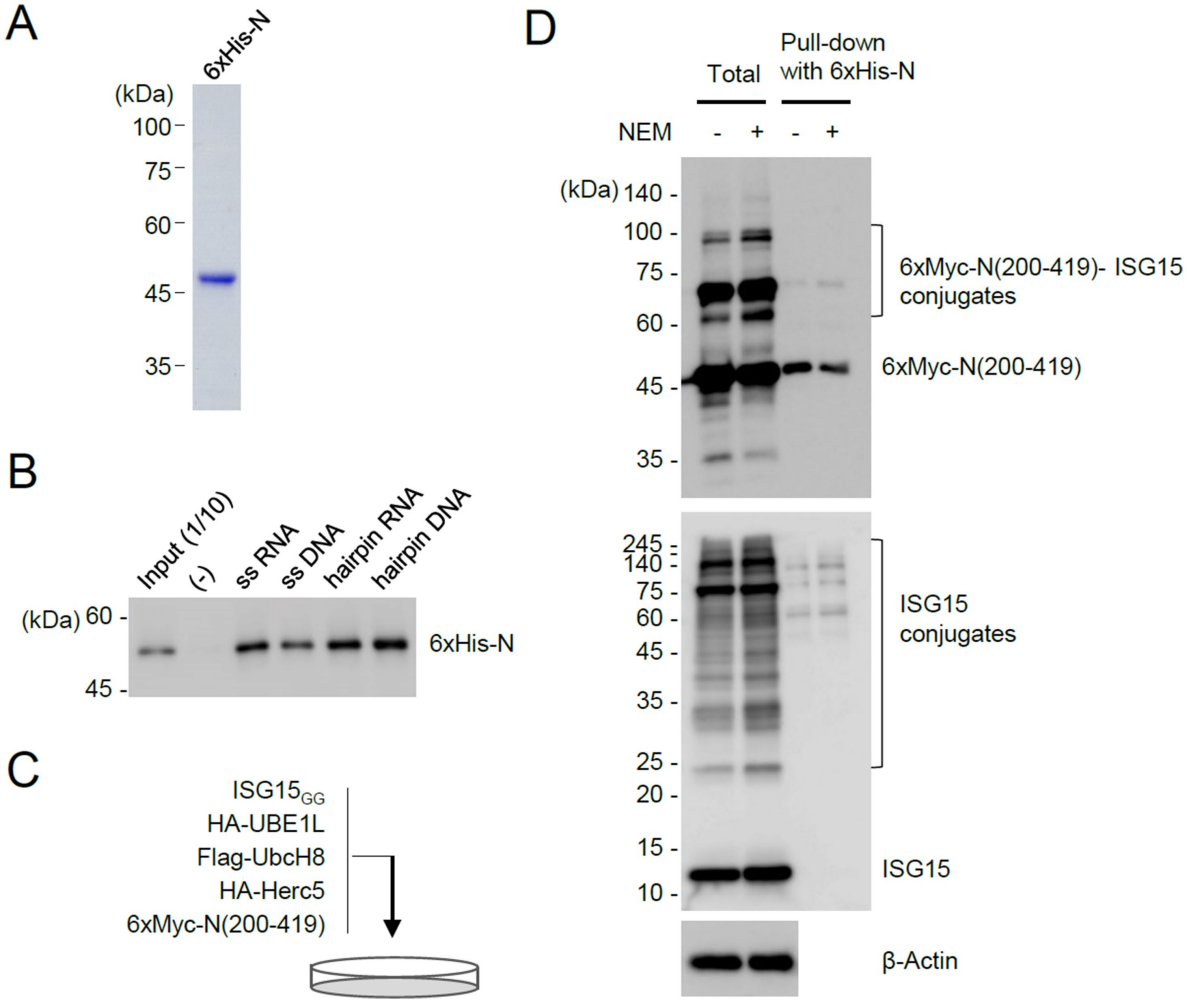
Pull-down assays demonstrating the inhibitory role of ISGylation for N dimerization. **(A)** N proteins with 6×His tags were produced in *E. coli* and purified using Ni-NTA resins. The purified 6×His-N proteins used for pull-down assays are shown by Coomassie Blue staining. **(B)** Bacterially purified 6×His-N (500 ng) were incubated with biotinylated nucleic acids (11.33 μM) (single-stranded RNA or DNA, or pre-incubated to form a hairpin structure) and immobilized on streptavidin beads. After washing, the bound proteins were eluted and determined by immunoblotting with anti-N antibody. Input (1/10) is 50 ng of purified 6×His-N. (−), no biotinylated oligonucleotides are added. **(C)** Production of unmodified and ISGylated N(200–419) proteins in cells. Plasmids expressing 6×Myc-N(200–419) (2 μg), HA-UBE1L (1.6 μg), Flag-UbcH8 (1.6 μg), HA-Herc5 (3.2 μg), and ISG15_GG_ (1.6 μg) were co-transfected into 293T cells in a 100-mm dish. Cells were untreated or treated with NEM (5 mM) for 30 min before harvesting. At 48 h after transfection, cell lysates were prepared. **(D)** Pull-down assays using 6×His-N. Unmodified and ISGylated N(200–419) proteins prepared from co-transfected cells were incubated with bacterially purified 6×His-N. N proteins with 6×His tags were then pulled down using Ni-NTA resins. As indicated, the input cell lysates and the samples obtained from the pull-down assay were analyzed by immunoblotting with anti-Myc, anti-ISG15, and anti-β-actin antibodies.

Considering that the C-terminal region of N containing the dimerization domain is ISGylated and that K261, a potential ISGylation site, is located at the dimer interface, we investigated whether ISGylation affects N protein dimerization. To address this question, we purified 6×His-tagged full-length N proteins in bacterial cells (Figure 5A) and used it in Ni^2+^–NTA pull-down assays. The N protein binds to single or double-stranded RNA and DNA *in vitro* (Zeng et al., 2020; Zhou R. et al., 2020; Zhao et al., 2024). The bacterially produced N proteins showed nucleic acids binding activity (Figure 5B). The unmodified and ISGylated N(200–419) proteins were produced in 293T cells after co-transfection of plasmids expressing 6×Myc-N(200–419), ISGylation enzymes, and ISG15_GG_ (Figure 5C). The purified 6×His-N were incubated with total cell lysates, and the 6×His-N proteins were pulled down with Ni^2+^–NTA-containing beads. The bound 6×Myc-N(200–419) proteins were eluted and subjected to immunoblotting. The results showed that 6×His-N more effectively interacted with unmodified 6×Myc-N(200–419) than its ISGylated forms, demonstrating that ISGylation of N inhibits protein dimerization (Figure 5D). Consistently, when we detected N oligomers in co-transfected cells using non-denaturing gel electrophoresis, the N(K261R) more effectively produced the oligomeric forms than the wild-type protein under the ISGylation conditions (Supplementary Figure S3).

### Enhanced resistance of a recombinant SARS-CoV-2 expressing N(K261R) to IFN-β treatment

**Figure 6:**
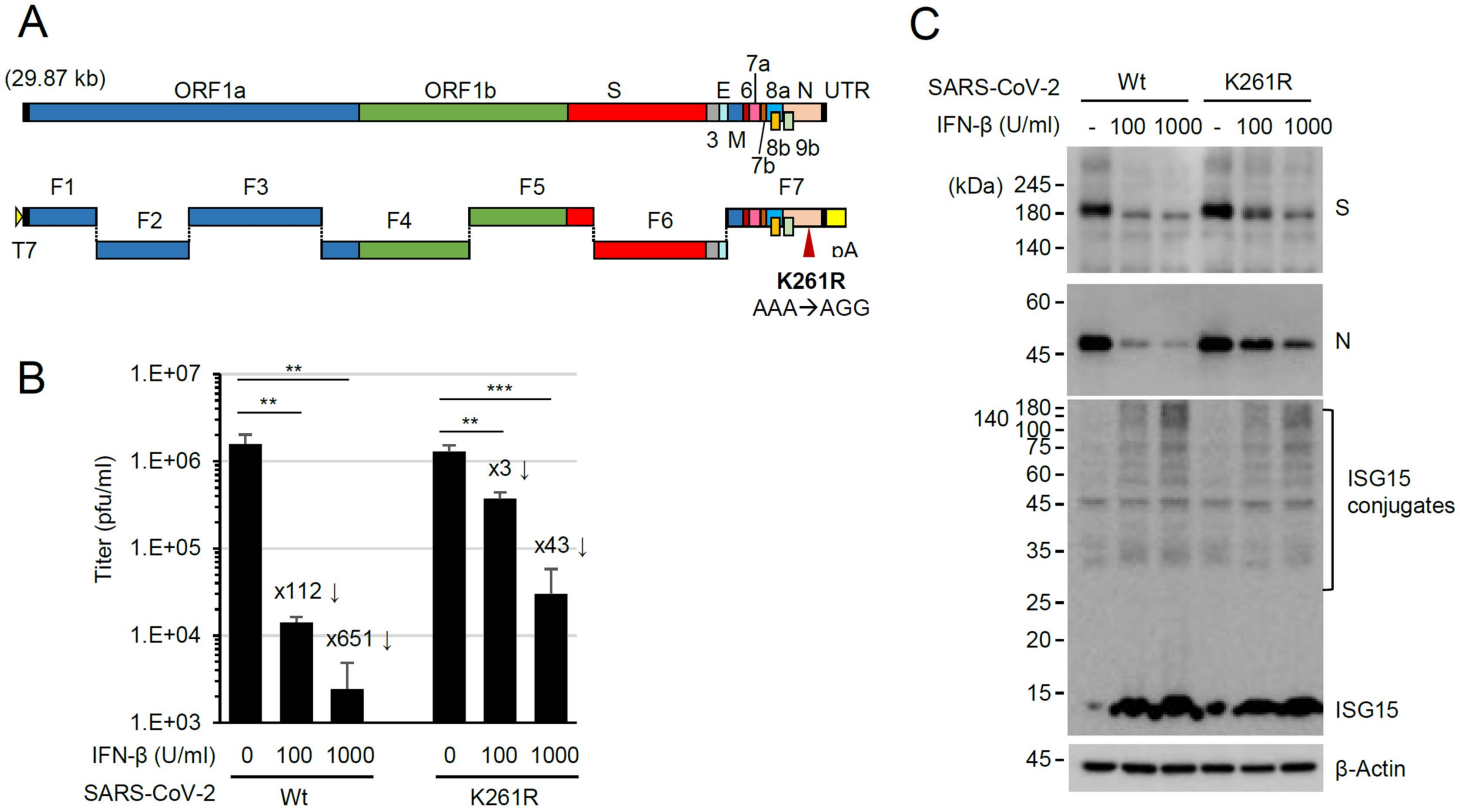
Enhanced resistance of the N(K261R) mutant virus to IFN-β treatment. **(A)** The seven plasmid-based system for the production of recombinant SARS-CoV-2. ORFs in the viral genome are indicated at the top, and the seven plasmids (F1 to F7) are shown underneath. F1 contains the T7 promoter, and F7 has poly(A) sequences. **(B,C)** Calu-3 cells in six-well plates were untreated or pretreated with 100 or 1,000 U/mL of IFN-β for 24 h and then infected with wild-type or N(K261R) mutant virus at an MOI of 0.1. At 24 h post-infection, the titers of the progeny virions in the culture supernatants were measured by plaque assays. Viral titers in three independent experiments are shown in graphs **(B)**. Samples were compared using Student’s *t*-test, and *p*-values (**p < 0.01 and ***p < 0.001) are indicated. The × numbers indicate the progeny virion production repression folds in IFN-β-treated cells over untreated cells. The levels of viral proteins (S and N) and ISGylated proteins in a representative experiment are shown by immunoblotting **(C)**.

To investigate the role of N ISGylation, a recombinant virus expressing N(K261R) was produced using a seven plasmid-based reverse genetic system of SARS-CoV-2 (Xie et al., 2020; Xie et al., 2021) and site-directed mutagenesis on the N gene of the F7 plasmid (Figure 6A). The whole genome sequencing results of the recombinant virus N(K261R) confirmed no other mutation in the recovered viral genome except the introduced K261R mutation (AAA to AGG). In our pull-down assays, the binding strength between the K261R mutant and the wild-type N and between the K261R mutants was similar to that between the wild-type N proteins, indicating that the K-to-R mutation did not affect the efficiency of N dimerization (Supplementary Figure S4). N is ubiquitinated through several lysine residues, but K261 does not appear to be involved (Stukalov et al., 2021; Mao et al., 2023; Zhao et al., 2023). We also observed that the overall ubiquitination patterns of N are similar between the wild-type and the K261R mutant in IFN-β treated cells (Supplementary Figure S5). When the production of progeny virions was measured by plaque assays, the N(K261R) mutant virus showed comparable growth to the wild-type virus in Calu-3 cells. However, when Calu-3 cells were treated with IFN-β (100 or 1,000 U/mL) for 24 h and then infected with the wild-type or N(K261R) mutant recombinant virus at an MOI of 1 and the production of progeny virions was determined by plaque assays, the growth of the N(K261R) mutant virus was less severely reduced compared to the wild-type virus (Figure 6B). This indicated that resistance to IFN-β was enhanced in the mutant virus compared to the parental virus. Consistent with this result, immunoblotting assays showed that the N(K261R) mutant virus expressed higher levels of the viral spike (S) and N proteins than its parental virus in IFN-β-treated cells, although a comparable increase of ISG15 and ISGylated protein levels was found in IFN-β-treated Calu-3 cells after infection with the wild-type or the N(K261R) mutant (Figure 6C).

### PLpro removes conjugated ISG15 from N

**Figure 7:**
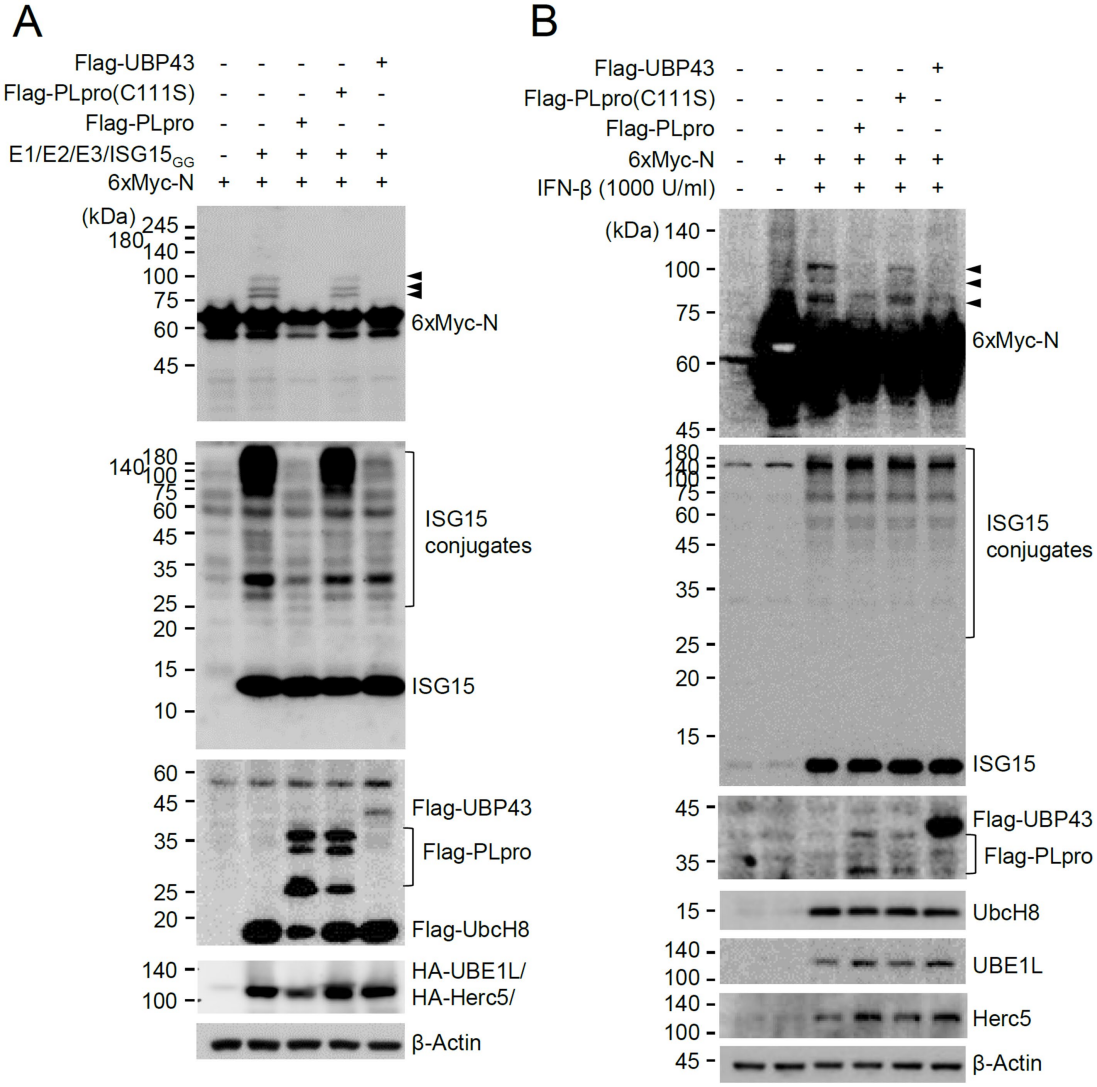
Cleavage of conjugated ISG15 from N by PLpro. **(A)** Plasmids expressing 6×Myc-N (0.25 μg) and plasmids expressing HA-UBE1L (E1, 0.2 μg), Flag-UbcH8 (E2, 0.2 μg), HA-Herc5 (E3, 0.4 μg), and ISG15_GG_ (0.2 μg) along with effector plasmids expressing wild-type or C111S mutant Flag-PLpro (0.25 μg) or Flag-UBP43 (0.25 μg), were co-transfected into 293T cells in six-well plates as indicated. At 48 h after transfection, total cell lysates were prepared and subjected to SDS-PAGE, and immunoblot assays were performed with anti-Myc, anti-ISG15, anti-Flag, anti-HA, and anti-β-Actin antibodies. Three ISGylated N protein bands are indicated with arrowheads. **(B)** HeLa cells were untreated or pretreated with IFN-β (1,000 U/mL) for 24 h and then transfected with a plasmid expressing 6×Myc-tagged N and plasmids expressing wild-type or mutant Flag-PLpro or Flag-UBP43 as in **(A)**. At 24 h after transfection, cell lysates were prepared and immunoblotted with antibodies for Myc, ISG15, Flag, UbcH8, UBE1L, Herc5, and β-Actin.

We next investigated whether viral PLpro encoded by NSP3 can deconjugate ISG15 from the ISG15-modified N. This was achieved by examining the effects of wild-type PLpro and its catalytic inactive mutant on ISGylation of N in co-transfection assays. PLpro inhibited the accumulation of ISGylated N proteins as effectively as UBP43, whereas the catalytic inactive mutant (C111S) did not (Figure 7A). When SRT-N was used in co-transfection assays, the single ISG15-modified form was effectively removed by PLpro (Supplementary Figure S6A). A similar loss of ISGylated N was also observed when intact NSP3 was used (Supplementary Figure S6B). These results demonstrated that PLpro can suppress the accumulation of ISGylated N proteins by cleaving conjugated ISG15 from N. We also tested whether PLpro can cleave ISG15 from N in IFN-β-treated cells. Transfection of PLpro or UBP43 did not markedly affect the overall levels of ISGylated proteins induced by IFN-β treatment, as only some of the cells were transfected; however, wild-type PLpro and UBP43 effectively inhibited the accumulation of ISGylated forms of co-transfected N or N(200–419), whereas the inactive PLpro mutant (C111S) did not (Figure 7B and Supplementary Figure S7). This indicated that PLpro can remove ISG15 from N in IFN-β-treated cells.

## Discussion

In this study, we demonstrate that the N protein of SARS-CoV-2 is targeted by ISG15 induced by type I IFNs, and N ISGylation suppresses viral growth by inhibiting N protein dimerization, which is essential for nucleocapsid formation and viral replication. Furthermore, we show that viral PLpro evades ISG15-mediated antiviral responses acting on this critical viral protein by removing conjugated ISG15 from N. The N protein was identified as a target of ISGylation in the screening of SARS-CoV-2-encoded proteins in cells that ectopically express ISG15, UBE1L (E1), UbcH8 (E2), and Herc5 (E3). In co-transfection assays, the use of N and ISG15 proteins with different tags produced a variation in the ISGylation patterns of N with one or more ISG15 moieties attached. We attribute this to structural changes in N and ISG15 proteins according to the tag. Our results indicate that Herc5 acts as a main E3 ligase for ISGylation because the other two human E3 ligases, EFP and HHARI, did not support N ISGylation. Importantly, ISGylation of N was demonstrated by the endogenous ISGylation machinery induced by IFN-β stimulation.

Detecting the ISGylated forms of N in SARS-CoV-2-infected cells was difficult because of the effective removal of conjugated ISG15 from N by viral PLpro. Previous studies showed an increase of ISGylated cellular proteins in SARS-CoV-2-infected cells after treatment with GRL0617, an inhibitor of PLpro (Shin et al., 2020; Liu et al., 2021). In these studies, cells were treated with the inhibitor simultaneously as virus infection. However, in that case, we observed that the expression of viral proteins was also inhibited, making detecting the ISGylated forms of N during virus infection difficult. However, a series of experimental results in this study support the idea that IFN-induced N ISGylation occurs in virus-infected cells. Different cell types utilize different activation patterns of cell type-specific STATs in response to type I IFNs, producing different impacts on cell subsets (van Boxel-Dezaire et al., 2006). Temporal conjugation of the bulky ISG15 molecule to the dimer interface of N may have a dominant negative effect on the structure and function of N in cell types exhibiting strong IFN responses.

Nucleocapsid is a multivalent RNA-binding protein critical for viral replication and genome packaging (Bai et al., 2021). N contains intrinsically disordered regions, including an N-terminal domain (NTD), a central Ser/Arg-rich linker domain, and a CTD, and conserved structured regions, including an RNA-binding domain and an oligomerization domain. We mapped the 257–361 region within the oligomerization domain involved in Herc5 binding and K261 as a potential ISGylation site that is necessary for efficient ISGylation of N. It has been reported that the cellular ISGylation machinery is induced upon SARS-CoV-2 infection, and the cellular proteins in innate immune responses, such as MDA5 and IRF3, are ISGylated, enhancing the cellular antiviral environment (Shin et al., 2020; Munnur et al., 2021). Our results demonstrate that the cellular ISGylation system also targets SARS-CoV-2 proteins to attenuate viral growth. Recent studies have demonstrated that N is a substrate for ubiquitination (Stukalov et al., 2021) and is poly-ubiquitinated at K375 by TRIM21, leading to proteasomal degradation (Mao et al., 2023) and at K143 by MARCH8, resulting in lysosomal degradation (Zhao et al., 2023). Our ubiquitination assays for N in IFN-β-treated cells suggest that ISGylation in K261 may not significantly affect the overall ubiquitination of N.

Our results showed that the ISGylation of N inhibited protein dimerization in a pull-down analysis using bacterially purified N and unmodified and ISGylated N(200–419). Given that N dimers are functional units for ribonucleoprotein assembly (Lutomski et al., 2021), these results demonstrate that ISGylation of N is a host immune response that inhibits SARS-CoV-2 replication by inhibiting N oligomerization and, consequently, the formation of nucleocapsids. Interestingly, ISGylation-mediated inhibition of viral protein oligomerization has been demonstrated. ISGylation of influenza A virus (IAV) NS1 interferes with dimerization and RNA binding (Tang et al., 2010) and blocks nuclear translocation by importin-*α* (Zhao et al., 2010). On the other hand, ISGylation of influenza B virus (IBV) NP blocks oligomerization and formation of viral ribonucleoprotein complexes (Zhao et al., 2016).

The recombinant SARS-CoV-2 expressing N(K261R) showed similar growth as its parental virus in Calu-3 cells, indicating that the substitution of Lys-261 to Arg does not affect the dimerization of N. However, the K261R virus became less sensitive to IFN-β treatment. This result supports that the IFN-induced ISGylation suppresses viral growth. In addition to nucleocapsid formation, SARS-CoV-2N plays various roles in regulating the cellular environment for virion production. N undergoes liquid–liquid phase separation (LLPS) to promote virion assembly (Carlson et al., 2020; Cascarina and Ross, 2020; Chen H. et al., 2020; Iserman et al., 2020; Perdikari et al., 2020; Savastano et al., 2020). N triggers NLRP3 inflammation activation (Pan et al., 2021) but inhibits pyroptosis and IL-1β release by blocking gasdermin D cleavage (Ma et al., 2021). N also inhibits the type I IFN response by reducing IFN production and the activation of ISGs (Chen K. et al., 2020; Li et al., 2020; Mu et al., 2020a) and acts as a suppressor of RNAi (Mu et al., 2020b). Therefore, it will be interesting to study whether ISGylation affects the functions of N in LLPS and the evasion of cellular antiviral responses.

SARS-CoV-2 PLpro has both deubiquitination and deISGylation activities. Our results showed that PLpro can remove ISG15 from ISG15-conjugated N proteins produced in cells ectopically overexpressing the ISGylation machinery and in cells expressing the endogenous ISGylation components induced by IFN-β stimulation. Notably, NSP3, which contains the PLpro domain, has been shown to interact with N (Jiang et al., 2021; Bessa et al., 2022; Li et al., 2023). The deISGylation activity of PLpro against N seems to promote viral replication by facilitating the formation of N dimers. PLpro has been shown to remove ISG15 from ISG15-conjugated IRF3 and MDA5 to reduce innate immune responses (Shin et al., 2020; Liu et al., 2021). SARS-CoV-2 PLpro has different binding modes toward ISG15 and a K48-linked ubiquitin dimer (K48-Ub2) (Wydorski et al., 2023). However, no PLpro mutation can completely separate the ISG15 and K48-Ub2 bindings of PLpro. Considering the low level of ISGylated N in virus-infected cells, to more accurately understand the effects of N ISGylation on viral infection, it will be necessary to produce a SARS-CoV-2 mutant with no deISGylation activity while maintaining deubiquitinating activity based on the structure of ISGylated N.

During the preparation of the manuscript, two studies reported that N is effectively ISGylated and PLpro inhibits it (Rhamadianti et al., 2024; Zhu et al., 2024). One study showed that other viral proteins, such as NSP7, NSP8, NSP10, NSP10, NSP12, and NSP16, are also ISGylated (Zhu et al., 2024). We initially screened the ISGylated form of viral proteins in total cell lysates, while this study detected ISGylated proteins using immunoprecipitated samples. Therefore, it is difficult to compare the results of our study with those of others directly. However, all studies show that N is the most effectively ISGylated protein among SARS-CoV-2 proteins, and PLpro inhibits N ISGylation. Although lysine residues identified as ISG15 receptors differ in studies, our findings complement these studies and confirm that SARS-CoV-2 has evolved to evade ISG15-mediated antiviral responses in hosts that target both viral and cellular proteins. We analyzed 15,613,092 nearly complete genome sequences of SARS-CoV-2 uploaded to GISAID as of Oct. 8, 2024. Mutations were observed at all ISGylation sites (K261, K266, K347, K355, K387, and K388) suggested by our study and others with mutation rates from 0.001 to 0.05%, indicating that some viruses might also have evolved to evade ISGylation by acquiring mutations at ISGylation sites.

## Data Availability

The original contributions presented in the study are included in the article/Supplementary material, further inquiries can be directed to the corresponding authors.

## References

[ref1] Arimoto K. I. Lochte S. Stoner S. A. Burkart C. Zhang Y. Miyauchi S.. (2017). STAT2 is an essential adaptor in USP18-mediated suppression of type I interferon signaling. Nat. Struct. Mol. Biol. 24, 279–289. doi: 10.1038/nsmb.3378, PMID: 28165510 PMC5365074

[ref2] Bai Z. Cao Y. Liu W. Li J. (2021). The SARS-CoV-2 Nucleocapsid protein and its role in viral structure, biological functions, and a potential target for drug or vaccine mitigation. Viruses 13:1115. doi: 10.3390/v13061115 34200602 PMC8227405

[ref3] Bessa L. M. Guseva S. Camacho-Zarco A. R. Salvi N. Maurin D. Perez L. M.. (2022). The intrinsically disordered SARS-CoV-2 nucleoprotein in dynamic complex with its viral partner nsp3a. Sci. Adv. 8:eabm4034. doi: 10.1126/sciadv.abm4034, PMID: 35044811 PMC8769549

[ref4] Bogunovic D. Byun M. Durfee L. A. Abhyankar A. Sanal O. Mansouri D.. (2012). Mycobacterial disease and impaired IFN-gamma immunity in humans with inherited ISG15 deficiency. Science 337, 1684–1688. doi: 10.1126/science.1224026, PMID: 22859821 PMC3507439

[ref5] Carlson C. R. Asfaha J. B. Ghent C. M. Howard C. J. Hartooni N. Safari M.. (2020). Phosphoregulation of phase separation by the SARS-CoV-2 N protein suggests a biophysical basis for its dual functions. Mol. Cell 80, 1092–1103.e4. doi: 10.1016/j.molcel.2020.11.025, PMID: 33248025 PMC7677695

[ref6] Cascarina S. M. Ross E. D. (2020). A proposed role for the SARS-CoV-2 nucleocapsid protein in the formation and regulation of biomolecular condensates. FASEB J. 34, 9832–9842. doi: 10.1096/fj.202001351, PMID: 32562316 PMC7323129

[ref7] Chen H. Cui Y. Han X. Hu W. Sun M. Zhang Y.. (2020). Liquid-liquid phase separation by SARS-CoV-2 nucleocapsid protein and RNA. Cell Res. 30, 1143–1145. doi: 10.1038/s41422-020-00408-2, PMID: 32901111 PMC7477871

[ref8] Chen K. Xiao F. Hu D. Ge W. Tian M. Wang W.. (2020). SARS-CoV-2 Nucleocapsid protein interacts with RIG-I and represses RIG-mediated IFN-β production. Viruses 13:47. doi: 10.3390/v13010047 33396605 PMC7823417

[ref9] Durfee L. A. Lyon N. Seo K. Huibregtse J. M. (2010). The ISG15 conjugation system broadly targets newly synthesized proteins: implications for the antiviral function of ISG15. Mol. Cell 38, 722–732. doi: 10.1016/j.molcel.2010.05.002, PMID: 20542004 PMC2887317

[ref10] Iserman C. Roden C. A. Boerneke M. A. Sealfon R. S. G. Mclaughlin G. A. Jungreis I.. (2020). Genomic RNA elements drive phase separation of the SARS-CoV-2 Nucleocapsid. Mol. Cell 80, 1078–1091.e6. doi: 10.1016/j.molcel.2020.11.041 33290746 PMC7691212

[ref11] Jiang Y. Tong K. Yao R. Zhou Y. Lin H. Du L.. (2021). Genome-wide analysis of protein-protein interactions and involvement of viral proteins in SARS-CoV-2 replication. Cell Biosci. 11:140. doi: 10.1186/s13578-021-00644-y 34294141 PMC8295636

[ref12] Kang J. A. Kim Y. J. Jeon Y. J. (2022). The diverse repertoire of ISG15: more intricate than initially thought. Exp. Mol. Med. 54, 1779–1792. doi: 10.1038/s12276-022-00872-3, PMID: 36319753 PMC9722776

[ref13] Kim Y. J. Kim E. T. Kim Y. E. Lee M. K. Kwon K. M. Kim K. I.. (2016). Consecutive inhibition of ISG15 expression and ISGylation by cytomegalovirus regulators. PLoS Pathog. 12:e1005850. doi: 10.1371/journal.ppat.1005850, PMID: 27564865 PMC5001722

[ref14] Kim D. Lee J. Y. Yang J. S. Kim J. W. Kim V. N. Chang H. (2020). The architecture of SARS-CoV-2 transcriptome. Cell 181, 914–921.e10. doi: 10.1016/j.cell.2020.04.011, PMID: 32330414 PMC7179501

[ref15] Lee J. M. Kang H. J. Lee H. R. Choi C. Y. Jang W. J. Ahn J. H. (2003). PIAS1 enhances SUMO-1 modification and the transactivation activity of the major immediate-early IE2 protein of human cytomegalovirus. FEBS Lett. 555, 322–328. doi: 10.1016/S0014-5793(03)01268-7 14644436

[ref16] Li J. Y. Liao C. H. Wang Q. Tan Y. J. Luo R. Qiu Y.. (2020). The ORF6, ORF8 and nucleocapsid proteins of SARS-CoV-2 inhibit type I interferon signaling pathway. Virus Res. 286:198074. doi: 10.1016/j.virusres.2020.198074, PMID: 32589897 PMC7309931

[ref17] Li P. Xue B. Schnicker N. J. Wong L. R. Meyerholz D. K. Perlman S. (2023). Nsp3-N interactions are critical for SARS-CoV-2 fitness and virulence. Proc. Natl. Acad. Sci. USA 120:e2305674120. doi: 10.1073/pnas.2305674120, PMID: 37487098 PMC10400999

[ref18] Liu G. Lee J. H. Parker Z. M. Acharya D. Chiang J. J. Van Gent M.. (2021). ISG15-dependent activation of the sensor MDA5 is antagonized by the SARS-CoV-2 papain-like protease to evade host innate immunity. Nat. Microbiol. 6, 467–478. doi: 10.1038/s41564-021-00884-1, PMID: 33727702 PMC8103894

[ref19] Lu R. Zhao X. Li J. Niu P. Yang B. Wu H.. (2020). Genomic characterisation and epidemiology of 2019 novel coronavirus: implications for virus origins and receptor binding. Lancet 395, 565–574. doi: 10.1016/S0140-6736(20)30251-8, PMID: 32007145 PMC7159086

[ref20] Lutomski C. A. El-Baba T. J. Bolla J. R. Robinson C. V. (2021). Multiple roles of SARS-CoV-2 N protein facilitated by Proteoform-specific interactions with RNA, host proteins, and convalescent antibodies. JACS Au 1, 1147–1157. doi: 10.1021/jacsau.1c00139 34462738 PMC8231660

[ref21] Ma J. Zhu F. Zhao M. Shao F. Yu D. Ma J.. (2021). SARS-CoV-2 nucleocapsid suppresses host pyroptosis by blocking Gasdermin D cleavage. EMBO J. 40:e108249. doi: 10.15252/embj.2021108249, PMID: 34296442 PMC8420271

[ref22] Malakhova O. A. Kim K. I. Luo J. K. Zou W. Kumar K. G. Fuchs S. Y.. (2006). UBP43 is a novel regulator of interferon signaling independent of its ISG15 isopeptidase activity. EMBO J. 25, 2358–2367. doi: 10.1038/sj.emboj.7601149 16710296 PMC1478183

[ref23] Mao S. Cai X. Niu S. Wei J. Jiang N. Deng H.. (2023). TRIM21 promotes ubiquitination of SARS-CoV-2 nucleocapsid protein to regulate innate immunity. J. Med. Virol. 95:e28719. doi: 10.1002/jmv.28719, PMID: 37185839

[ref24] Meuwissen M. E. Schot R. Buta S. Oudesluijs G. Tinschert S. Speer S. D.. (2016). Human USP18 deficiency underlies type 1 interferonopathy leading to severe pseudo-TORCH syndrome. J. Exp. Med. 213, 1163–1174. doi: 10.1084/jem.20151529, PMID: 27325888 PMC4925017

[ref25] Morales D. J. Lenschow D. J. (2013). The antiviral activities of ISG15. J. Mol. Biol. 425, 4995–5008. doi: 10.1016/j.jmb.2013.09.041 24095857 PMC4090058

[ref26] Mu J. Fang Y. Yang Q. Shu T. Wang A. Huang M.. (2020a). SARS-CoV-2 N protein antagonizes type I interferon signaling by suppressing phosphorylation and nuclear translocation of STAT1 and STAT2. Cell Discov 6:65. doi: 10.1038/s41421-020-00208-3, PMID: 32953130 PMC7490572

[ref27] Mu J. Xu J. Zhang L. Shu T. Wu D. Huang M.. (2020b). SARS-CoV-2-encoded nucleocapsid protein acts as a viral suppressor of RNA interference in cells. Sci. China Life Sci. 63, 1413–1416. doi: 10.1007/s11427-020-1692-1, PMID: 32291557 PMC7154568

[ref28] Munnur D. Teo Q. Eggermont D. Lee H. H. Y. Thery F. Ho J.. (2021). Altered ISGylation drives aberrant macrophage-dependent immune responses during SARS-CoV-2 infection. Nat. Immunol. 22, 1416–1427. doi: 10.1038/s41590-021-01035-8, PMID: 34663977

[ref29] Pan P. Shen M. Yu Z. Ge W. Chen K. Tian M.. (2021). SARS-CoV-2 N protein promotes NLRP3 inflammasome activation to induce hyperinflammation. Nat. Commun. 12:4664. doi: 10.1038/s41467-021-25015-6 34341353 PMC8329225

[ref30] Perdikari T. M. Murthy A. C. Ryan V. H. Watters S. Naik M. T. Fawzi N. L. (2020). SARS-CoV-2 nucleocapsid protein phase-separates with RNA and with human hnRNPs. EMBO J. 39:e106478. doi: 10.15252/embj.2020106478, PMID: 33200826 PMC7737613

[ref31] Perng Y. C. Lenschow D. J. (2018). ISG15 in antiviral immunity and beyond. Nat. Rev. Microbiol. 16, 423–439. doi: 10.1038/s41579-018-0020-5, PMID: 29769653 PMC7097117

[ref001] Rhamadianti A. F. Abe T. Tanaka T. Ono C. Katayama H. Makino Y.. (2024). SARS-CoV-2 papain-like protease inhibits ISGylation of the viral nucleocapsid protein to evade host anti-viral immunity. J. Virol. e0085524. 39120134 10.1128/jvi.00855-24 PMC11406913

[ref32] Sarkar L. Liu G. Gack M. U. (2023). ISG15: its roles in SARS-CoV-2 and other viral infections. Trends Microbiol. 31, 1262–1275. doi: 10.1016/j.tim.2023.07.006, PMID: 37573184 PMC10840963

[ref33] Savastano A. Ibáñez De Opakua A. Rankovic M. Zweckstetter M. (2020). Nucleocapsid protein of SARS-CoV-2 phase separates into RNA-rich polymerase-containing condensates. Nat. Commun. 11:6041. doi: 10.1038/s41467-020-19843-1, PMID: 33247108 PMC7699647

[ref34] Schlee M. (2013). Master sensors of pathogenic RNA – RIG-I like receptors. Immunobiology 218, 1322–1335. doi: 10.1016/j.imbio.2013.06.007, PMID: 23896194 PMC7114584

[ref35] Shin D. Mukherjee R. Grewe D. Bojkova D. Baek K. Bhattacharya A.. (2020). Papain-like protease regulates SARS-CoV-2 viral spread and innate immunity. Nature 587, 657–662. doi: 10.1038/s41586-020-2601-5, PMID: 32726803 PMC7116779

[ref36] Speer S. D. Li Z. Buta S. Payelle-Brogard B. Qian L. Vigant F.. (2016). ISG15 deficiency and increased viral resistance in humans but not mice. Nat. Commun. 7:11496. doi: 10.1038/ncomms11496, PMID: 27193971 PMC4873964

[ref37] Stukalov A. Girault V. Grass V. Karayel O. Bergant V. Urban C.. (2021). Multilevel proteomics reveals host perturbations by SARS-CoV-2 and SARS-CoV. Nature 594, 246–252. doi: 10.1038/s41586-021-03493-4, PMID: 33845483

[ref38] Swaim C. D. Canadeo L. A. Monte K. J. Khanna S. Lenschow D. J. Huibregtse J. M. (2020). Modulation of extracellular ISG15 signaling by pathogens and viral effector proteins. Cell Rep. 31:107772. doi: 10.1016/j.celrep.2020.107772, PMID: 32553163 PMC7297157

[ref39] Tang Y. Zhong G. Zhu L. Liu X. Shan Y. Feng H.. (2010). Herc5 attenuates influenza A virus by catalyzing ISGylation of viral NS1 protein. J. Immunol. 184, 5777–5790. doi: 10.4049/jimmunol.0903588 20385878

[ref40] Turner D. L. Weintraub H. (1994). Expression of achaete-scute homolog 3 in Xenopus embryos converts ectodermal cells to a neural fate. Genes Dev. 8, 1434–1447. doi: 10.1101/gad.8.12.1434, PMID: 7926743

[ref41] Van Boxel-Dezaire A. H. Rani M. R. Stark G. R. (2006). Complex modulation of cell type-specific signaling in response to type I interferons. Immunity 25, 361–372. doi: 10.1016/j.immuni.2006.08.014 16979568

[ref42] V'kovski P. Kratzel A. Steiner S. Stalder H. Thiel V. (2021). Coronavirus biology and replication: implications for SARS-CoV-2. Nat. Rev. Microbiol. 19, 155–170. doi: 10.1038/s41579-020-00468-6 33116300 PMC7592455

[ref43] Wu F. Zhao S. Yu B. Chen Y. M. Wang W. Song Z. G.. (2020). A new coronavirus associated with human respiratory disease in China. Nature 579, 265–269. doi: 10.1038/s41586-020-2008-3, PMID: 32015508 PMC7094943

[ref44] Wydorski P. M. Osipiuk J. Lanham B. T. Tesar C. Endres M. Engle E.. (2023). Dual domain recognition determines SARS-CoV-2 PLpro selectivity for human ISG15 and K48-linked di-ubiquitin. Nat. Commun. 14:2366. doi: 10.1038/s41467-023-38031-5, PMID: 37185902 PMC10126577

[ref45] Xie X. Lokugamage K. G. Zhang X. Vu M. N. Muruato A. E. Menachery V. D.. (2021). Engineering SARS-CoV-2 using a reverse genetic system. Nat. Protoc. 16, 1761–1784. doi: 10.1038/s41596-021-00491-8, PMID: 33514944 PMC8168523

[ref46] Xie X. Muruato A. Lokugamage K. G. Narayanan K. Zhang X. Zou J.. (2020). An infectious cDNA clone of SARS-CoV-2. Cell Host Microbe 27, 841–848.e3. doi: 10.1016/j.chom.2020.04.004, PMID: 32289263 PMC7153529

[ref47] Zeng W. Liu G. Ma H. Zhao D. Yang Y. Liu M.. (2020). Biochemical characterization of SARS-CoV-2 nucleocapsid protein. Biochem. Biophys. Res. Commun. 527, 618–623. doi: 10.1016/j.bbrc.2020.04.136 32416961 PMC7190499

[ref48] Zhang X. Bogunovic D. Payelle-Brogard B. Francois-Newton V. Speer S. D. Yuan C.. (2015). Human intracellular ISG15 prevents interferon-alpha/beta over-amplification and auto-inflammation. Nature 517, 89–93. doi: 10.1038/nature13801 25307056 PMC4303590

[ref49] Zhao C. Hsiang T. Y. Kuo R. L. Krug R. M. (2010). ISG15 conjugation system targets the viral NS1 protein in influenza A virus-infected cells. Proc. Natl. Acad. Sci. USA 107, 2253–2258. doi: 10.1073/pnas.0909144107, PMID: 20133869 PMC2836655

[ref50] Zhao C. Sridharan H. Chen R. Baker D. P. Wang S. Krug R. M. (2016). Influenza B virus non-structural protein 1 counteracts ISG15 antiviral activity by sequestering ISGylated viral proteins. Nat. Commun. 7:12754. doi: 10.1038/ncomms12754, PMID: 27587337 PMC5025834

[ref51] Zhao Y. Sui L. Wu P. Li L. Liu L. Ma B.. (2023). EGR1 functions as a new host restriction factor for SARS-CoV-2 to inhibit virus replication through the E3 ubiquitin ligase MARCH8. J. Virol. 97:e0102823. doi: 10.1128/jvi.01028-23 37772822 PMC10653994

[ref52] Zhao H. Syed A. M. Khalid M. M. Nguyen A. Ciling A. Wu D.. (2024). Assembly of SARS-CoV-2 nucleocapsid protein with nucleic acid. Nucleic Acids Res. 52, 6647–6661. doi: 10.1093/nar/gkae256, PMID: 38587193 PMC11194069

[ref53] Zhou P. Yang X. L. Wang X. G. Hu B. Zhang L. Zhang W.. (2020). A pneumonia outbreak associated with a new coronavirus of probable bat origin. Nature 579, 270–273. doi: 10.1038/s41586-020-2012-7, PMID: 32015507 PMC7095418

[ref54] Zhou R. Zeng R. Von Brunn A. Lei J. (2020). Structural characterization of the C-terminal domain of SARS-CoV-2 nucleocapsid protein. Mol Biomed 1:2. doi: 10.1186/s43556-020-00001-4, PMID: 34765991 PMC7406681

[ref002] Zhu J. Liu G. Sayyad Z. Goins C. M. Stauffer S. R. Gack M. U. (2024). ISGylation of the SARS-CoV-2 N protein by HERC5 impedes N oligomerization and thereby viral RNA synthesis. J. Virol, e0086924. 39194248 10.1128/jvi.00869-24 PMC11406920

[ref55] Zhu N. Zhang D. Wang W. Li X. Yang B. Song J.. (2020). A novel coronavirus from patients with pneumonia in China, 2019. N. Engl. J. Med. 382, 727–733. doi: 10.1056/NEJMoa2001017, PMID: 31978945 PMC7092803

