# Supplementary material for: Suppression of SARS-CoV-2 nucleocapsid protein dimerization by ISGylation and its counteraction by viral PLpro
Source: Front Microbiol. 2024 Oct 24;15:1490944. doi: 10.3389/fmicb.2024.1490944 (PMC11540652; doi:10.3389/fmicb.2024.1490944)
Supplement: Supplementary file 1 [file Data_Sheet_1.DOCX]

**Supplemental Material**

**Suppression of SARS-CoV-2 nucleocapsid protein dimerization by ISGylation and its counteraction by viral PLpro**

Wonjin Bang^1,†^, Jaehyun Kim^2,†^, Kanghun Seo^1^, Jihyun Lee^1^, Ji Ho Han^2^, Daegyu Park^1^, Jae Hwan Cho^2^, Donghyuk Shin^3^, Kyun-Hwan Kim^4^, Moon Jung Song^2,*^, and Jin-Hyun Ahn^1,*^

^1^Department of Microbiology, Sungkyunkwan University School of Medicine, Suwon 16419, ^2^Department of Biotechnology, College of Life Sciences and Biotechnology, Korea University, Seoul 02841, ^3^Department of Systems Biology, Yonsei University, Seoul 03722, ^4^Department of Precision Medicine, Sungkyunkwan University School of Medicine, Suwon 16419, Republic of Korea.

Running title: ISGylation of SARS-CoV-2 N

*Corresponding authors: Jin-Hyun Ahn, Department of Microbiology, Sungkyunkwan University School of Medicine, 2066 Seoburo, Jangangu, Suwon 16419, Republic of Korea. Tel: +82-31-299-6222, E-mail: [jahn@skku.edu](mailto:jahn@skku.edu); Moon Jung Song, Department of Biotechnology, College of Life Sciences and Biotechnology, Korea University, Seoul 02841, Republic of Korea. Tel: +82-2-3290-3019, E-mail: moonsong@korea.ac.kr

^†^Wonjin Bang and Jaehyun Kim contributed equally to this work.

Figs S1-S7


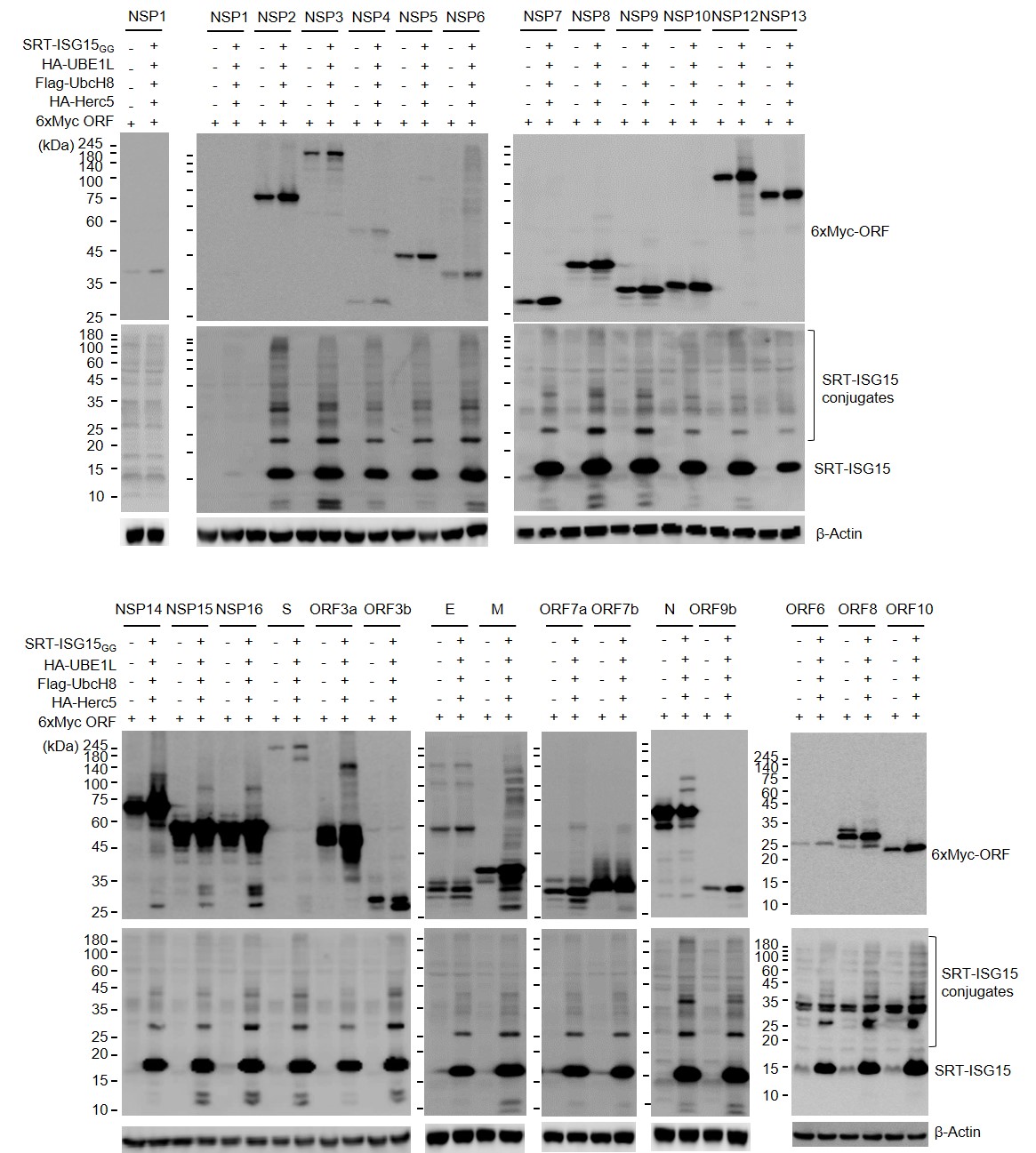


**Fig. S1. Identification of N as a substrate for ISGylation.** 293T cells in six-well plates were co-transfected with plasmids expressing 6×Myc-SARS-CoV-2 proteins (0.25 μg), HA-UBE1L (E1, 0.2 μg), Flag-UbcH8 (E2, 0.2 μg), HA-Herc5 (E3, 0.4 μg), and SRT-ISG15_GG_ (0.2 μg) as indicated. At 48 h after transfection, total cell lysates were prepared and subjected to SDS-PAGE, followed by immunoblot assays with anti-Myc, anti-SRT, anti-ISG15, or anti-β-Actin antibodies. A representative result of the experimental sets is shown.


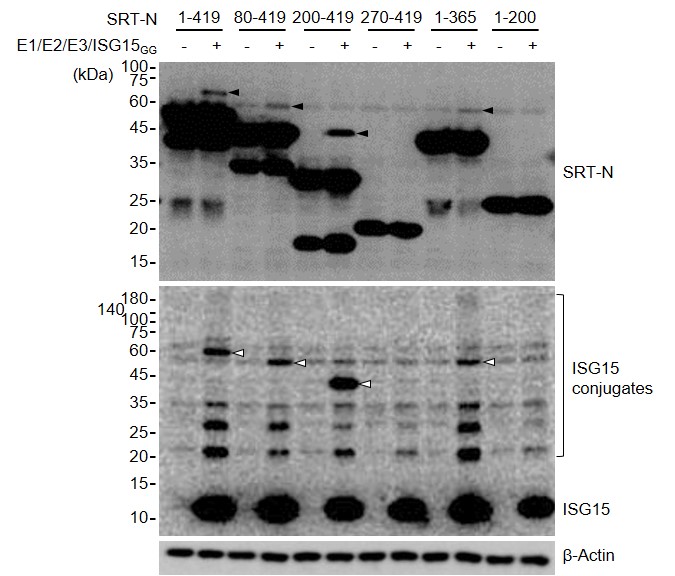


**Fig. S2. Identification of the N domains required for ISGylation with SRT-N.** Plasmids expressing SRT-N or its truncated forms (0.25 μg), HA-UBE1L (E1, 0.2 μg), Flag-UbcH8 (E2, 0.2 μg), HA-Herc5 (E3, 0.4 μg), and ISG15_GG_ (0.2 μg) were co-transfected into 293T cells in six-well plates. At 48 h after transfection, total cell lysates were prepared and subjected to SDS-PAGE, followed by immunoblot assays with anti-Myc, anti-ISG15, and anti-β-Actin antibodies. The positions of SRT-N proteins with ISG15 moieties in anti-SRT blot are indicated (black arrowheads). The corresponding ISG15-conjugated N proteins detected in the anti-ISG15 blot are indicated (white arrowheads).


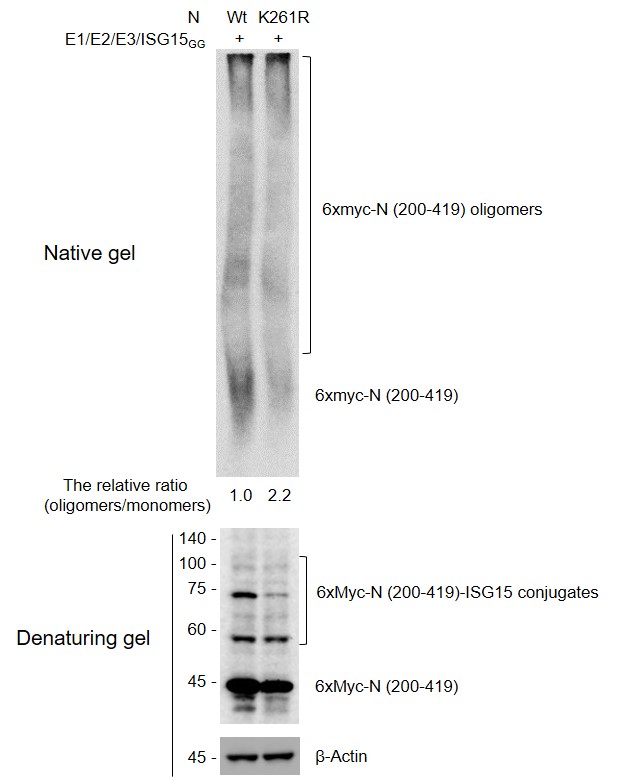


**Fig. S3. Comparison of N oligomerization between the wild-type and K261R mutant proteins under the ISGylation conditions.** 293T cells in six-well plates were co-transfected with plasmids expressing 6×Myc-N(200-419) (wild-type or K261R mutant) (0.25 μg), HA-UBE1L (0.2 μg), Flag-UbcH8 (0.2 μg), HA-Herc5 (0.4 μg) and ISG15_GG_ (0.2 μg). At 48 h after transfection, total cell lysates were lysed with buffer (50 mM Tris-HCl, pH 7.5, RT, 150 mM NaCl, 50 mM NaF, 5 mM Sodium pyrophosphate, 5% Glycerol, and 0.1% Triton X-100) and subjected to native gel (4%) electrophoresis (top) or SDS-PAGE (10%) (middle and bottom), and immunoblot assays were performed with anti-Myc and anti-β-Actin antibodies. The levels of β-actin are shown as a loading control. The position for oligomeric forms of N(200-419) is indicated. The relative ratio of oligomeric forms to monomeric forms is also shown as numbers.


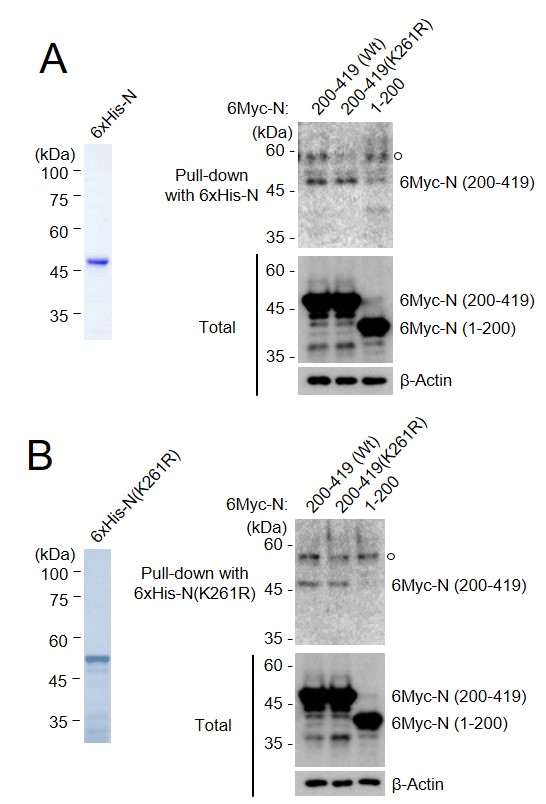


**Fig. S4. Pull-down assays using 6×His-N to compare the dimerization efficiency of the wild-type and the K261R mutant N proteins.** The wild-type or K261R mutant 6×Myc-N(200-419) or 6×Myc-N(1-200) proteins in transfected cell lysates were incubated with bacterially purified 6×His-N (A) or 6×His-N(K261R) (B), followed by pull-down using Ni-NTA resins. As indicated, the input cell lysates and the pulled-down samples were analyzed by immunoblotting with anti-Myc and anti-β-actin antibodies. The purified 6×His-N proteins used for pull-down assays are shown by Coomassie Blue staining. Circles, non-specific bands detected in pulled-down samples


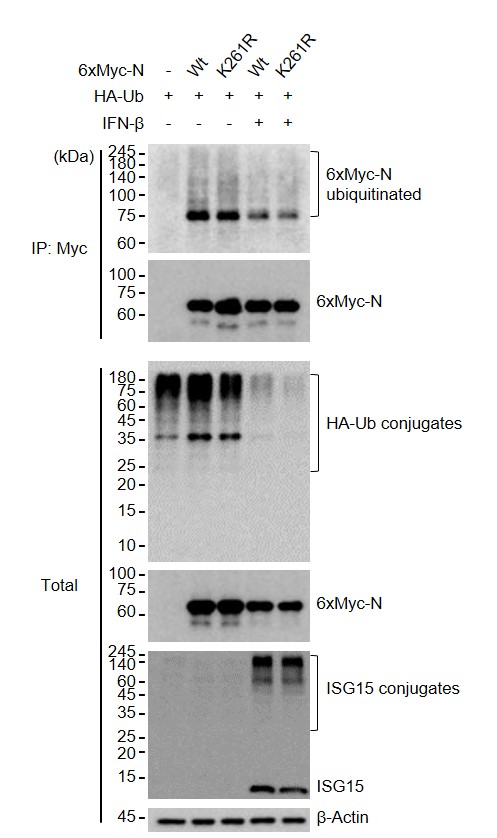


**Fig. S5. Effect of IFN-β-induced ISGylation on ubiquitination of N.** HeLa cells (2.5 × 10^5^) in six-well plates were untreated or pretreated with IFN-β (1000 U/ml) for 24 h, followed by co-transfection with plasmids expressing 6xMyc-N (wild-type or K261R) or HA-ubiquitin (Ub) with or without IFN-β treatment, as indicated. At 24 h after co-transfection, cells were harvested, and cell pellets were resuspended with 2% SDS lysis buffer containing protease inhibitors (Sigma) and boiled for 10 min. Cell lysates were diluted ten-fold with Co-IP buffer, and the clarified cell lysates were immunoprecipitated with anti-Myc antibodies and then immunoblotted with anti-HA or anti-Myc antibodies. Total cell lysates were also immunoblotted with anti-HA, anti-Myc, or anti-ISG15 antibodies. Levels of β-actin are shown as a loading control.


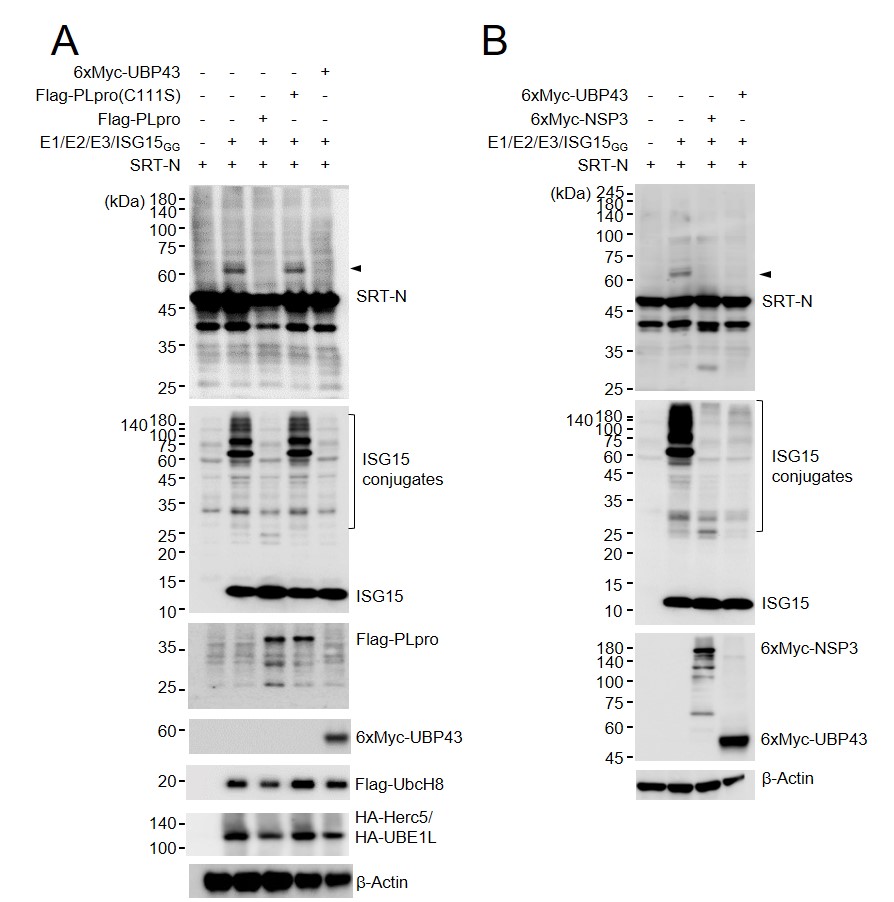


**Fig. S6. ISG15 cleavage assays from SRT-N by PLpro.** (A and B) Plasmids expressing SRT-N (0.25 μg), and plasmids expressing HA-UBE1L (E1; 0.2 μg), Flag-UbcH8 (E2; 0.2 μg), HA-Herc5 (E3; 0.4 μg) and ISG15_GG_ (0.2 μg) along with effector plasmids expressing wild-type or C111S mutant Flag-PLpro (0.25 μg), or Flag-UBP43 (0.25 μg) (A), or with plasmids expressing Flag-UBP43 (0.25 μg) or Myc-NSP3 (0.25 μg) (B) were co-transfected into 293T cells in six-well plates as indicated. At 48 h after transfection, total cell lysates were prepared and subjected to SDS-PAGE, and immunoblot assays were performed with anti-SRT, anti-ISG15, anti-Flag, and anti-HA, or anti-Myc, and anti-β-Actin antibodies. The ISGylated N protein bands are indicated with arrowheads.


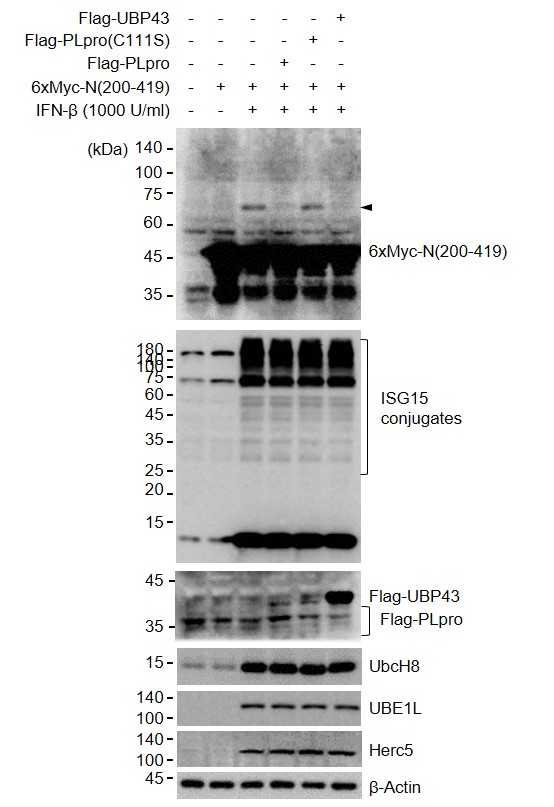


**Fig. S7.** **Cleavage of conjugated ISG15 from N by PLpro in IFN-β-treated cells.** HeLa cells were untreated or pretreated with IFN-β (1,000 U/ml) for 24 h and then transfected with a plasmid expressing 6×Myc-tagged N and plasmids expressing wild-type or mutant Flag-PLpro or Flag-UBP43. At 24 h after transfection, cell lysates were prepared and immunoblotted with antibodies for Myc, ISG15, Flag, UbcH8, UBE1L, Herc5, and β-Actin. The ISGylated N protein band is indicated with an arrowhead.
